# Comparing PVP and Polymeric Micellar Formulations of a PEGylated Photosensitizing Phthalocyanine by NMR and Optical Techniques

**DOI:** 10.1021/acs.molpharmaceut.3c00306

**Published:** 2023-07-26

**Authors:** Lea P. Gergely, Çiğdem Yüceel, Ümit İşci, Florentin S. Spadin, Lukas Schneider, Bernhard Spingler, Martin Frenz, Fabienne Dumoulin, Martina Vermathen

**Affiliations:** † Department of Chemistry, Biochemistry and Pharmaceutical Sciences, University of Bern, Bern 3012, Switzerland; ‡ Department of Chemical Engineering, Gebze Technical University, Gebze 41400 Kocaeli, Turkey; § Department of Chemistry, Gebze Technical University, Gebze 41400 Kocaeli, Turkey; ∥ Marmara University, Faculty of Technology, Department of Metallurgical & Materials Engineering, Istanbul 34722, Turkey; ⊥ Institute of Applied Physics, University of Bern, Bern 3012, Switzerland; # Department of Chemistry, University of Zurich, Zurich 8057, Switzerland; ¶ Faculty of Engineering and Natural Sciences, Biomedical Engineering Department, Acıbadem Mehmet Ali Aydınlar University, Ataşehir, Istanbul 34752, Turkey

**Keywords:** phthalocyanine, photosensitizer, drug delivery, polymer micelles, PVP, triblock copolymers, Kolliphor RH40, NMR spectroscopy, fluorescence spectroscopy

## Abstract

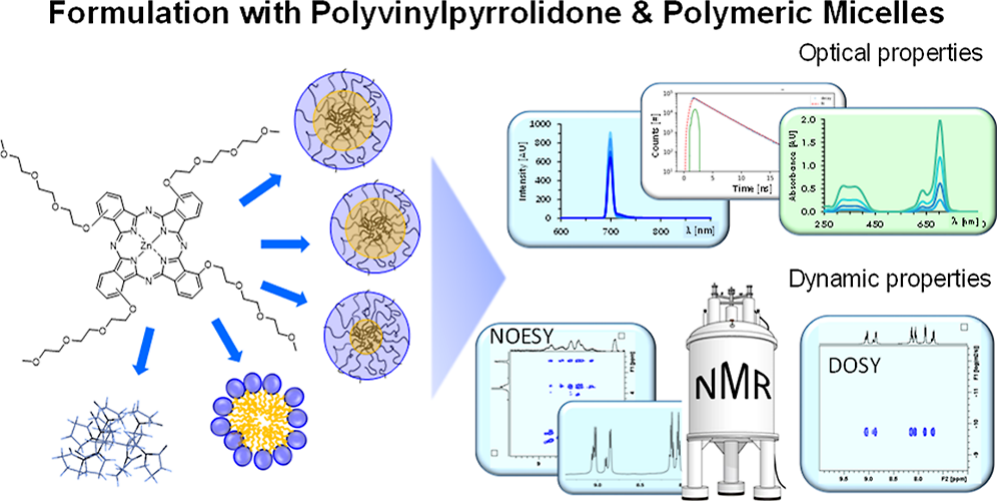

Phthalocyanines are ideal candidates as photosensitizers for photodynamic therapy (PDT) of cancer due to their favorable chemical and photophysical properties. However, their tendency to form aggregates in water reduces PDT efficacy and poses challenges in obtaining efficient forms of phthalocyanines for therapeutic applications. In the current work, polyvinylpyrrolidone (PVP) and micellar formulations were compared for encapsulating and monomerizing a water-soluble zinc phthalocyanine bearing four non-peripheral triethylene glycol chains (**Pc1**). ^1^H NMR spectroscopy combined with UV–vis absorption and fluorescence spectroscopy revealed that **Pc1** exists as a mixture of regioisomers in monomeric form in dimethyl sulfoxide but forms dimers in an aqueous buffer. PVP, polyethylene glycol castor oil (Kolliphor RH40), and three different triblock copolymers with varying proportions of polyethylene and polypropylene glycol units (termed P188, P84, and F127) were tested as micellar carriers for **Pc1**. ^1^H NMR chemical shift analysis, diffusion-ordered spectroscopy, and 2D nuclear Overhauser enhancement spectroscopy was applied to monitor the encapsulation and localization of **Pc1** at the polymer interface. Kolliphor RH40 and F127 micelles exhibited the highest affinity for encapsulating **Pc1** in the micellar core and resulted in intense **Pc1** fluorescence emission as well as efficient singlet oxygen formation along with PVP. Among the triblock copolymers, efficiency in binding and dimer dissolution decreased in the order F127 > P84 > P188. PVP was a strong binder for **Pc1**. However, **Pc1** molecules are rather surface-attached and exist as monomer and dimer mixtures. The results demonstrate that NMR combined with optical spectroscopy offer powerful tools to assess parameters like drug binding, localization sites, and dynamic properties that play key roles in achieving high host–guest compatibility. With the corresponding adjustments, polymeric micelles can offer simple and easily accessible drug delivery systems optimizing phthalocyanines’ properties as efficient photosensitizers.

## 1 Introduction

Among the numerous classes of molecular photosensitizers used in anti-cancer photodynamic therapy (PDT), phthalocyanines have the intrinsic advantage to absorb at far-red wavelengths in the first phototherapeutic window, hence offering a deep penetration of light into biological tissues while avoiding the excitation of endogenous chromophores. Together with their chemical versatility,^1^ this makes phthalocyanines ideal photosensitizers for PDT.^2,3^ To improve phthalocyanines’ biocompatibility, many substitution patterns have been used to make them water soluble,^4^ and various drug delivery systems have been developed for poorly water soluble or organosoluble phthalocyanines.^5,6^ Phthalocyanines can be covalently combined into water-dispersible nanoparticles of various types, such as organosilica nanoparticles,^7^ polymeric materials,^8−10^ and targeting units, such as nanobodies.^11,12^

**Figure 1:**
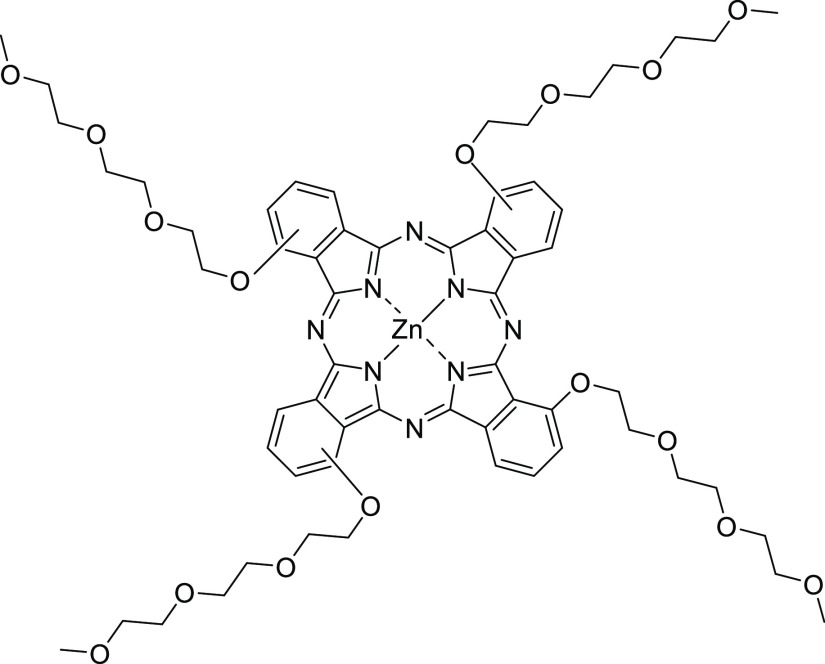
Structure of the 1, 8(11), 15(18), 22(25)-tetrakis-(2-(2-(2-methoxyethoxy)ethoxy)ethoxy)-Zn(II) phthalocyanine (**Pc1**).

However, non-covalent formulation is often acknowledged as a relatively simpler system. A pioneer example was the liposomal formulation of unsubstituted zinc phthalocyanine (ZnPc), known as CGP55847, which reached advanced clinical trials.^13^ Since then, formulations of photosensitizing phthalocyanines in self-emulsifying drug delivery systems^14^ like the polyethylene glycol (PEG) esters of castor oil (Kolliphor EL and Kolliphor RH, former synonyms: Cremophor EL and Cremophor RH, respectively) have been widely used.^15−17^ Their weak toxicities^18,19^ have not been an issue for these applications. Micellar formulations consisting of triblock copolymers with PEG–polypropylene glycol (PPG)–PEG units, among others, are also gaining momentum.^20−22^ More recently, poly(*N*-vinylpyrrolidone) (PVP) has proved to be an effective formulation material for photosensitizers,^23−26^ including phthalocyanines.^23^ Simple dilutions of dimethyl sulfoxide (DMSO) stock solutions of a phthalocyanine with culture medium are less universal but used when relevant. In an attempt to explore the effect of these different modes of administration on photodynamic investigations, we previously studied the in vitro photodynamic efficiency of the water-soluble PEGylated Zn(II) phthalocyanine (**Pc1**, Figure 1) from water stock solutions and DMSO stock solutions and after PVP formulations.^27^ Moreover, we recently have shown the importance of choosing suitable encapsulating polymeric materials to prevent photosensitizer self-aggregation, thus optimizing its photoproperties and finally the photodynamic outcome.^28^ Nuclear magnetic resonance (NMR) spectroscopy has proven to be an especially powerful tool for such studies.^29−31^ Besides structural information, it provides insight into dynamic processes like diffusion, exchange, and molecular mobility as well as into intermolecular interactions with atomic resolution. In the current study, NMR-spectroscopic methods were combined with optical methods including UV–vis absorption, steady-state and time-resolved fluorescence spectroscopies to assess the capability of five different polymeric materials for the physical encapsulation of **Pc1**. The objectives pursued, i.e., a measure of phthalocyanine monomerization and enhancement of light absorption and emission properties, are inevitable prerequisites to make the corresponding phthalocyanine a promising candidate as an efficient PDT drug.

## 2 Materials and Methods

### 2.1 Materials

1(4), 8(11), 15(18), 22(25)-(2-(2-(2-methoxyethoxy)ethoxy)ethoxy)-Zn(II) phthalocyanine (**Pc1**, MW = 1226.64 g/mol) was synthesized as previously described.^27,32^ PVP (average MW = 10 kDa), PVP (average MW = 40 kDa), PEG–PPG–PEG triblock copolymers Kolliphor P188 (P188, average MW = 8.4 kDa), Synperonic PE/P84 (P84, average MW = 4.2 kDa), and Pluronic F-127 (F127, average MW = 12.6 kDa), the PEG ester of hydrogenated castor oil Kolliphor RH 40 (RH40, average MW = 2.5 kDa), and methylene blue (MB) were purchased from Sigma-Aldrich. Deuterated solvents, DMSO-*d*_6_ (99.95%) and D_2_O (99.9%), used for the NMR measurements were purchased from Eurisotop (St.-Aubin Cedex, France) and Deutero GmbH (Kastelaun, Germany), respectively. Phosphate-buffered saline [(PBS), 50 mM, pH = 7.3] was prepared by mixing aliquots of 50 mM solutions of KH_2_PO_4_ and Na_2_HPO_4_ (provided by Sigma-Aldrich) in D_2_O or H_2_O containing 0.9% NaCl. The human epithelioid cervix carcinoma HeLa cell line was purchased from Merck and cultured in minimum essential medium (MEM, Gibco). Paraformaldehyde [(PFA), 16%, Electron Microscopy Sciences] was used for cell fixation.

### 2.2 Sample Preparation

#### 2.2.1 Solutions for NMR Spectroscopy

A 10 mM stock solution of **Pc1** in DMSO-*d*_6_ was prepared and used for structural characterization of the regioisomers as well as for the preparation of a 1 mM solution of **Pc1** in D_2_O-based PBS and further dilutions in the absence and presence of the polymeric carriers. Stock solutions of PVP, P188, P84, F127, and RH40 were prepared in D_2_O-based PBS at concentrations of 10–40 mM. For complete dissolving, the polymer/PBS mixtures were vortexed, sonicated, and left standing overnight at room temperature. Polymer solutions of 10 mM were used for direct NMR investigation in the absence of the phthalocyanine. Encapsulation of **Pc1** into the polymeric carriers was done by mixing aliquots of **Pc1** in DMSO-*d*_6_ and the corresponding polymer stock solution to reach final concentrations of 1 mM of **Pc1** and 10 mM of each polymer. The mixtures were vortexed and transferred to NMR tubes. Additional solutions of **Pc1** in the absence and presence of polymers were prepared in the same way at phthalocyanine concentrations of 0.1, 0.2, 0.5, and 2 mM and polymer concentrations each of 10 mM in D_2_O-based PBS.

#### 2.2.2 Solutions for UV–Vis and Fluorescence Spectroscopies

All solutions were prepared in DMSO or in H_2_O-based PBS on the day of measurements. For UV–vis spectroscopy, concentrations of **Pc1** were 1, 2.5, 5, and 10 μM and polymer concentrations were each 2 mM. For fluorescence spectroscopy, concentrations of **Pc1** were 0.1, 0.25, 0.5, and 1 μM and polymer concentrations were each 2 mM.

### 2.3 Methods

#### 2.3.1 NMR Spectroscopy

The 1D and 2D NMR spectroscopy measurements were carried out with a Bruker Avance II spectrometer operating at a resonance frequency of 500.13 MHz (11.74 T) for ^1^H and 125.76 MHz for ^13^C equipped with a 5 mm broadband inverse (BBI) probe-head with a z-gradient (5.35 G/mm) coil. The NMR spectroscopy measurements at different concentrations of **Pc1** (0.1–2 mM) were run on a Bruker AVANCE NEO spectrometer at 500.13 MHz/125.76 MHz (for ^1^H/^13^C) using a 1.7 mm triple resonance (^1^H, ^13^C, ^31^P) inverse (TXI) microprobe head with a z-gradient coil. All measurements were performed at ambient temperature. Acquisition and processing of the spectra were done using the Bruker software TopSpin version 4.1.4 and 4.0.9, respectively. Diffusion-ordered spectroscopy (DOSY) data were processed using the Bruker software Dynamics Center, Version 2.6.2.

1D ^1^H NMR spectra were recorded using the “*zg*” pulse sequence from the Bruker pulse program library acquiring 64 scans with a 90°-pulse over a spectral width of 15 ppm with 64 K data points, a relaxation delay of 6 s, and an acquisition time of 4.37 s. The spectra were processed by applying exponential multiplication with a line-broadening factor of 1 Hz, Fourier transformation, phasing, and baseline correction with a first-degree polynomial. ^1^H DOSY was performed using a stimulated echo pulse sequence with bipolar gradients, longitudinal eddy current delay, spoil gradients, and presaturation of the residual water resonance (“*ledbpcpgp2scpr*” from the Bruker pulse program library). The gradient strength was linearly incremented in 32 steps from 5 to 95% of its full strength. The diffusion time (Δ) was set to 200 ms, and the gradient length (δ) to 4.6 ms to achieve optimal signal attenuation over the whole gradient ramp. Each increment was acquired with 32 scans, 8 K data points, and a spectral width of 15 ppm. The single spectra were processed applying exponential multiplication with a line-broadening factor of 1 Hz, Fourier transform, phasing, and baseline correction (5th degree polynomial). Mono-exponential fitting, according to the Stejskal–Tanner equation,^33^ was performed for selected resonances to obtain the 2D DOSY spectra. The 2D ^1^H^1^H nuclear Overhauser enhancement spectroscopy (NOESY) experiments were performed using the phase-sensitive pulse programs “*noesygpphpp*” (for **Pc1** in DMSO-*d*_6_) and “*noesygpphprf1*” (for all samples in D_2_O-PBS) from the Bruker pulse program library with gradient pulses and presaturation of the residual water resonance during the relaxation delay on the F1 channel. The mixing time was set at 500 ms (for **Pc1** in DMSO-*d*_6_) and 100 ms (for all samples in D_2_O-PBS) and the relaxation delay as 1 s. For each increment, 8–16 scans were acquired over a spectral width of 15 ppm with 2048 data points in F2 and 128–512 data points in F1. For structural analysis of pure **Pc1** in DMSO-*d*_6_, 2D ^1^H^1^H COSY and 2D ^1^H^13^C HSQC spectra were recorded using the pulse programs “*cosygpppqf*” and “*hsqcedetgpsisp2.2*,” respectively, with multiplicity editing (both from the Bruker pulse program library).

#### 2.3.2 UV–Vis Spectroscopy

UV–vis absorption spectra were recorded on a double-beam Thermo Scientific Evolution 201 UV–visible Spectrophotometer operated by the “Insight 2” Thermo Scientific software. The samples were transferred to quartz cuvettes of 1 cm optical path length with reduced volume (1 mL). Absorption spectra were measured at room temperature over a wavelength range of 250–900 nm at a scan rate of 1000 nm/min in 1 nm increments. The slit width was 2 nm, and the integration time was 0.06 s. Absorption spectra were plotted using Excel (2016, Microsoft) software. Molar absorption coefficients ε were determined from linear regressions from plots of the absorbance A (at λ_max_) versus the concentration c of **Pc1** according to the Lambert–Beer equation (*A* = ε·*c*·*l*) in Excel.

#### 2.3.3 Fluorescence Spectroscopy

Fluorescence spectra were recorded on a Cary Eclipse G9800A Fluorescence spectrophotometer (Agilent Technologies) operated by the “Cary Eclipse Scan Application” software (version 1.2(147), Agilent Technologies). The samples were transferred to QS quartz glass high-precision cells of 1 cm path length and a sample volume of 2 mL. Fluorescence emission was measured at room temperature over a wavelength range of 600–900 nm at a scan rate of 600 nm/min and an excitation wavelength of λ_ex_ = 350 nm. Excitation and emission slit widths were both 5 nm. Emission spectra were plotted using Excel.

#### 2.3.4 Fluorescence Lifetime Imaging Microscopy

For fluorescence lifetime imaging microscopy (FLIM), HeLa cells were grown on coverslips in 12-well plates in 1 mL MEM to reach a density of 360 cells/mm^2^ after the incubation time. The cells were incubated with either 5 μM **Pc1** in PBS alone, or combined with 16.7 μM PVP or 3 mM RH40 for 24 h and kept in dark. Subsequently, the cells were collected and washed with PBS, fixed with 150 μL of 4% PFA for 15 min, and washed twice with PBS. Then, the coverslips were flipped onto a microscopy slide with PBS as the mounting medium and sealed with nail polish. The samples were kept in the refrigerator at 6 °C until measurements were recorded. Fluorescence lifetime measurements were also performed on the **Pc1** solutions used for cell incubation.

Fluorescence lifetime measurements were conducted in time-correlated single-photon counting mode using a Zeiss LSM 10 confocal laser scanning microscope adapted for fluorescence lifetime imaging using a FLIM upgrade kit (Becker & Hickl PZ-FLIM-110). Fluorescence excitation was conducted using a pulsed diode laser (PicoQuant LDH-C 400) with a wavelength of 405 nm and a pulse length of 130 ps. Emission was measured from 590 nm to 720 nm. Measurements were conducted at room temperature at a pulse repetition rate of 20 MHz, yielding an average photon counting rate of 10^5^ s^–1^. Measurements were analyzed using SPCImageNG software (Becker & Hickl). For model-based evaluation, a biexponential model was used with lifetimes τ_1_ and τ_2_ constrained to the interval [0.2–99] ns and τ_2_ additionally constrained to at least a factor 2 larger than τ_1_. The instrument response function was estimated from the decay curve by differentiation of the rising edge and therefore did not need to be measured.

#### 2.3.5 Encapsulation Efficiency and Drug Loading

The determination of encapsulation efficiency (EE) and drug loading (DL) were performed by separating the fraction of non-entrapped drug after ultrafiltration. For this, Amicon Ultra Centrifugal Filters (regenerated cellulose) for 0.5 mL sample volumes and a molecular weight cutoff (MWCO) of 10 kDa (Merck Millipore Ltd. Tullagreen, Ireland) were used. Solutions of **Pc1** mixed with the five different carriers were prepared in PBS-D_2_O as described in 2.2.1 yielding final concentrations of 1 mM **Pc1** and 10 mM polymer (molar ratio **Pc1**:polymer = 1:10). From each solution, 100 μL was transferred to the filter reservoir and centrifuged at 14,000 g for 30 min. A solution of 1 mM **Pc1** in PBS was equally treated and used as reference. All filtration permeates were diluted by a factor of 100, and absorption spectra were recorded under the same conditions as described in 2.3.2. The amount of **Pc1** recovered from the filtrate was calculated from the absorption at 660 and 700 nm and averaged. The percentages of EE and DL were calculated according to eqs 1 and 2, respectively^34^
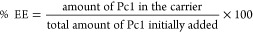
1
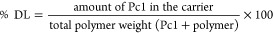
2

#### 2.3.6 Singlet Oxygen Quantum Yields

The singlet oxygen quantum yields (Φ_Δ_) of **Pc1-PBS**, **Pc1-P188**, **Pc1-P84**, **Pc1-F127**, **Pc1-RH40**, and **Pc1-PVP** were determined with the aid of a previously reported setup.^35,36^ The samples were prepared in D_2_O-based PBS, and the solutions were placed in a 10 × 4 mm 114F-10-40 fluorescence 1 mL quartz glass cuvette (Hellma Analytics, Germany). The cuvette was oriented in a way that the light path equals to 10 mm. Two different concentrations (in the range 3–16 μM) were prepared for each measured sample while the maximum absorption intensity of the highest concentrated sample did at no point exceed 1.0 A. For this, the samples were placed in a temperature-controlled UV/Vis cuvette (CUV–UV/VIS-TC-ABS, Avantes, the Netherlands) and cooled to 20 °C, whereby the temperature control was achieved with the Q-Blue software (Quantum Northwest, USA). An AvaLight-HAL-S-Mini lamp (Avantes) connected through an FC-UVIR600-1-BX optical fiber cable (Avantes) to the sample compartment was used as the light source for the UV–vis measurements. The same type of cable was used to connect the sample compartment to the AvaSpec-ULS2048CL-EVO-RS detector (Avantes) used for the UV–vis measurements.

Identical sample solutions as applied for the UV–vis measurements were used for ^1^O_2_ emission spectroscopy, which was conducted with the same custom-built setup that is based on a method described in literature.^37^ Excitation was performed using a high-power FC-LED-690M light source (690 nm, Prizmatix Ltd., Israel). The light source was connected through an FC-IR1000-2 optical fiber cable (Avantes) to the sample compartment in a way that the excitation path equals to 4 mm. The intensity of the light source was measured to be 9.0 mW/cm^2^ at the position of the cuvette. The excitation power was measured using a S310C thermal power sensor connected to a PM100USB power and energy meter interface (Thorlabs, Germany). The connection piece used to insert the SMA connector into the sample compartment was replaced by an in-house custom-built connection piece that allows the fiber to be inserted at 2.0 cm from the cuvette. The AvaSpec-NIR256-1.7TEC detector (Avantes) used for the measurements was set to 0 °C and connected to the sample compartment (20 °C) with the same optical fiber cable as used for the UV–vis measurements. ^1^O_2_ emission spectra were collected at a 90° angle with respect to the excitation beam from 1050 to 1500 nm after two measurement runs. Every measurement run consisted of five averaged measurements, while each lasted 9 s. All spectra were recorded using the AvaSoft 8.14 software (Avantes) and were further processed using the software Excel (Microsoft, USA) and Origin 2022 (OriginLab, USA).

The Φ_Δ_ of the standard MB in D_2_O-based PBS (Φ_Δ_ = 0.60 ± 0.02) was first determined by applying eq 3 and the Φ_Δ_ value for MB in D_2_O reported in literature (Φ_Δ_ = 0.52).^38,39^ Then, eq 3 was used to calculate the Φ_Δ_ values of **Pc1-PBS**, **Pc1-P188**, **Pc1-P84**, **Pc1-F127**, **Pc1-RH40**, and **Pc1-PVP** with the aid of the previously determined Φ_Δ_ value of MB in D_2_O-based PBS. All samples were measured with the same excitation light source, in the same solvent, and with the same number of measurement runs.
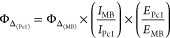
3In eq 3, the subscript “Pc1” designates the corresponding sample, and “MB” denotes the standard MB. “*I*” is the rate of light absorption calculated as overlap of the absorption spectrum of either sample or MB and the emission spectrum of the LED light source (*I*_0_). The absorption intensity depends exponentially on the absorbance A (eq 4). “*E*” is the integrated emission peak of ^1^O_2_ at around 1270 nm. For these ^1^O_2_ emission spectra, the integrated values were obtained by applying a manual background correction with the Origin 2022 software.
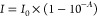
4

Equation 3 can be rewritten as
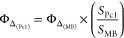
5In eq 5, “*S*” designates the slope when “*E*” is plotted against “*I*” for the two measured concentrations, with a fixed intercept at 0. The results are given as Φ_Δ_ values, while the limit of detection of the applied setup is estimated to be 0.10. Errors of the Φ_Δ_ values were calculated by error propagation from the standard errors of “*S*_Pc1_” and “*S*_MB_”.

## 3 Results and Discussion

### 3.1 Regioisomers of **Pc1**

**Figure 2:**
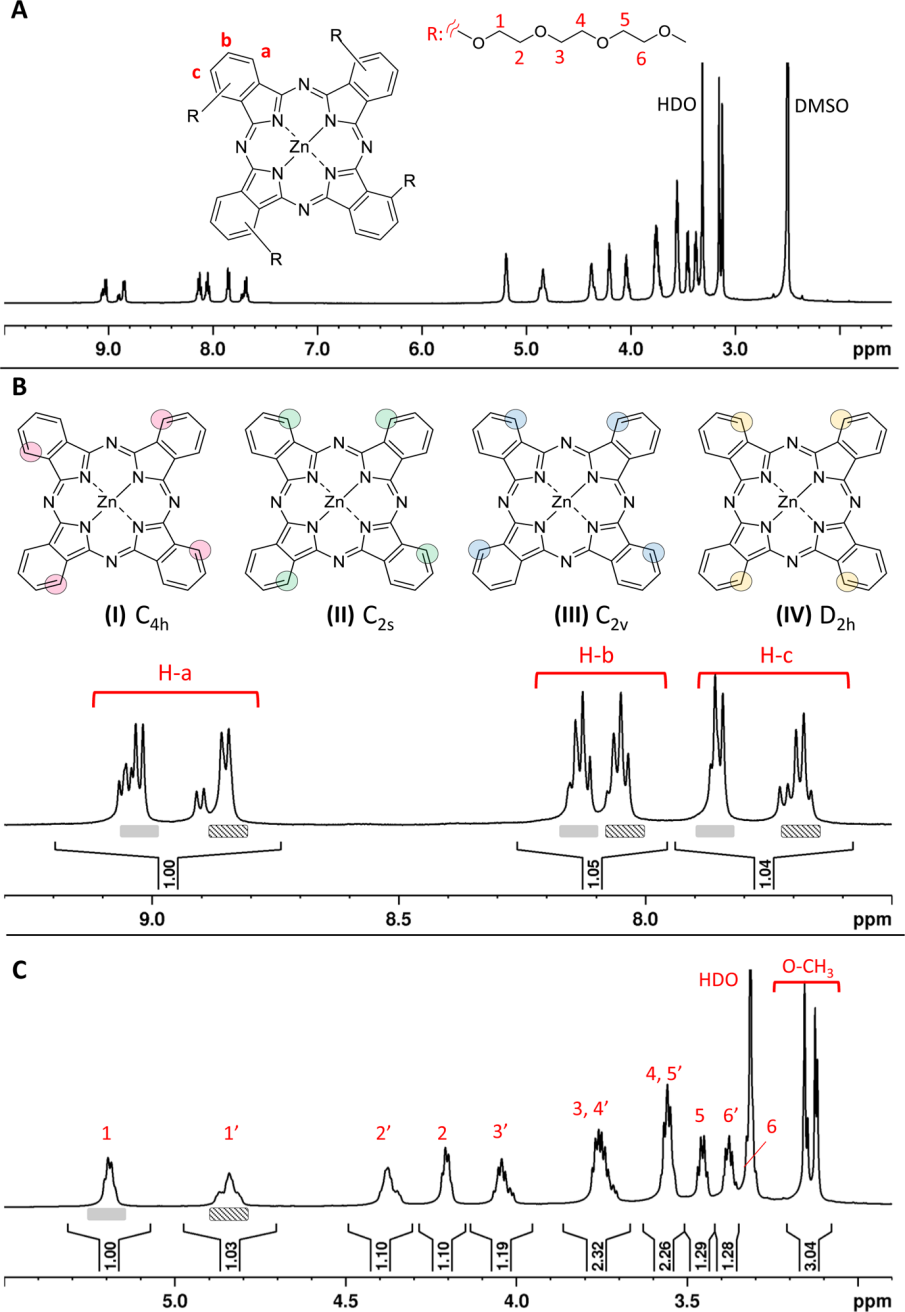
^1^H NMR spectrum of **Pc1** in DMSO-*d*_6_. (A) Spectral overview; (B) structures of the four potential regioisomers and spectral region between 7.4 and 9.3 ppm showing the aromatic resonances; (C) spectral region between 2.8 and 5.5 ppm showing the resonances of the TriEG chains.

The ^1^H NMR spectrum of **Pc1** dissolved in DMSO is shown in Figure 2A–C. Resonance assignments were based on additional 2D NMR spectra (Figures S1–S3) and literature data.^40,41^ The triethylene glycol (TriEG) −CH_2_ resonances are spread between 3.3 and 5.5 ppm (Figure 2C) with the most downfield shifted ones deriving from the ethylene glycol (EG) groups closest to the phthalocyanine macrocycle as was confirmed by NOE data (Figure S3). The existence of non-equivalent resonances for each of the aromatic protons in positions a, b, and c (Figure 2B) as well as for the EG-CH_2_ protons (Figure 2C) indicates the coexistence of isomers with different relative positions of the TriEG substituents. Synthesis of **Pc1** via the cyclotetramerization of the corresponding mono-TriEG-substituted phthalonitriles can theoretically result in the formation of four different regioisomers^40−42^ as delineated in Figure 2B. For α-tetra-alkoxy-substituted phthalocyanines, the statistically favored isomer (**II**) is typically the prevalent one formed at about 40–50%, whereas the amount of isomer (**IV**) is very low due to sterical hindrance.^40,43^ For **Pc1**, two sets of non-equivalent EG resonances are observed at a roughly equal molar ratio. TriEG chains facing each other as in isomers (**II**)–(**IV**) give rise to downfield shifted resonances^41^ (peaks 1–6, Figure 2C) whereas TriEG chains distant from each other as in isomers (**I**)–(**III**) have slightly different chemical shift values marked with 1′–6′ in Figure 2C. The corresponding aromatic proton signals of the rings bearing either facing or distant TriEG chains are marked in Figure 2 and were assigned based on COSY and NOE spectra (Figures S1 and S3). The molar ratio of EG resonances, the presence of at least five resolved doublets for H-a, and the statistically expected regioisomer ratios suggest the existence of two main isomers, namely the least symmetric isomers (**II**) and (**III**). However, since the single resonances of the regioisomers overlap, their assignment remains ambiguous.

### 3.2 Behavior of Pc1 in Aqueous PBS solution

**Figure 3:**
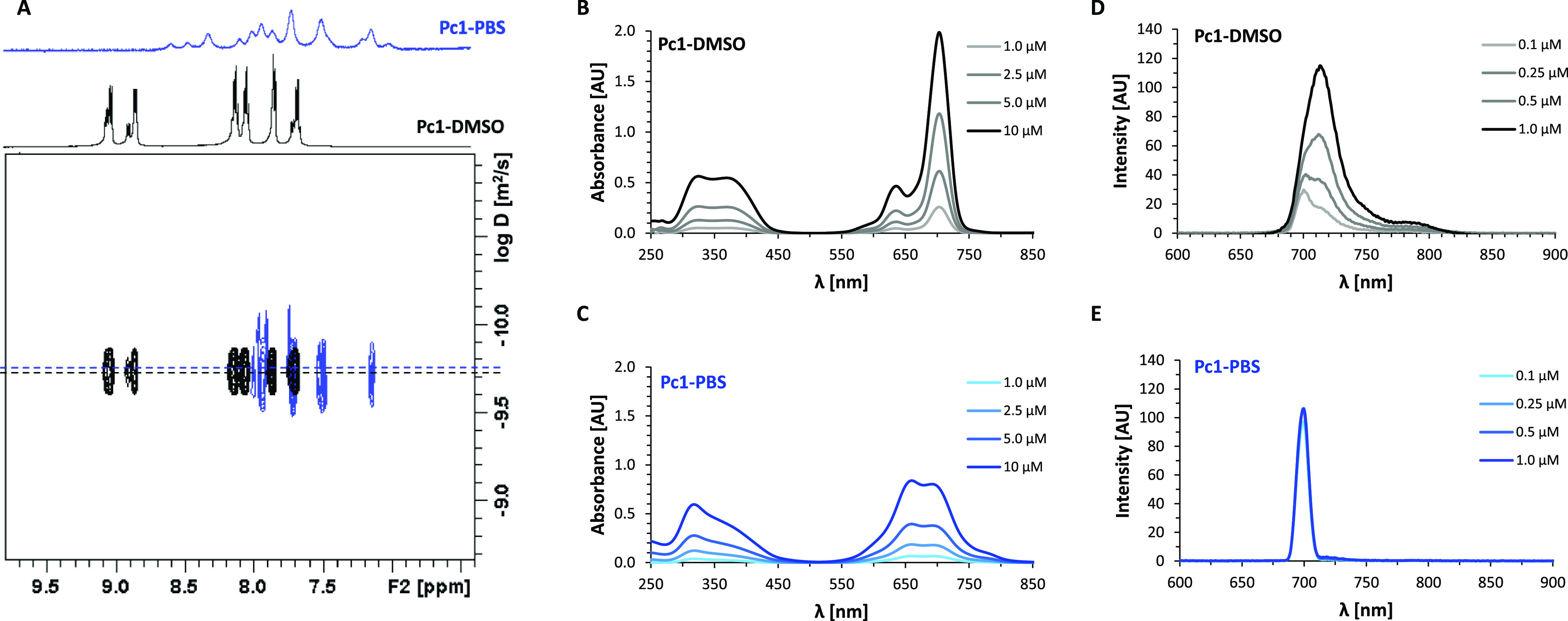
(A) ^1^H DOSY NMR spectrum of **Pc1** in DMSO-*d*_6_ (black) and D_2_O-based PBS (blue) with projections of the 1D ^1^H NMR spectra displaying the aromatic region. (B) UV–vis spectra of **Pc1** at concentrations of 1, 2.5, 5, and 10 μM dissolved in DMSO and (C) in PBS. (D) Fluorescence spectra of **Pc1** at concentrations of 0.1, 0.25, 0.5, and 1 μM dissolved in DMSO and (E) in PBS.

**Pc1** is water-soluble owing to its four TriEG substituents at the non-peripheral α-positions. However, NMR-spectroscopic and photospectrometric data reveal that, despite its water solubility, aggregates exist in aqueous buffer solutions. In Figure 3A, an overlay of the ^1^H DOSY spectra of **Pc1** either dissolved in DMSO (**Pc1-DMSO**, black) or in PBS (**Pc1-PBS**, blue) are shown for the aromatic region along with the corresponding 1D ^1^H projection spectra. The diffusion coefficient *D* is given on the *y*-axis in logarithmic scale for each proton resonance with sufficient resonance intensity in the spectral chemical shift dimension on the *x*-axis. The well-resolved ^1^H NMR resonances of **Pc1-DMSO** indicate that it forms monomers in DMSO (Figure 2 and projection spectrum in Figure 3A). On the other hand, resonance line-broadening and upfield shifts of **Pc1-PBS** point to the formation of aggregates in PBS-D_2_O^44^ (Figure 3A, projection spectrum). Since the *D*-values of **Pc1-DMSO** and **Pc1-PBS** are very similar, the phthalocyanine most likely forms dimers rather than higher aggregates in PBS. This appears feasible if the difference in viscosity η of the solvents is taken into account, which is approximately a factor of 2 for DMSO-*d*_6_ (η = 2.195 mPa*s) and D_2_O (η = 1.097 mPa*s) at room temperature.^45^ According to the Stokes–Einstein eq 6, the diffusion coefficient *D* is inversely correlated to the viscosity η of the diffusion medium and to the molecular size given as hydrodynamic radius *r*_H_ (with *k*_B_ being the Boltzmann constant and *T* being the temperature).
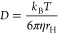
6

Accordingly, the *D*-value will only differ by a factor of 2 for dimers under the same diffusion conditions compared to that for monomers.

In Figure 3B,C, the UV–vis absorption spectra of **Pc1-DMSO** and **Pc1-PBS** are shown, respectively, for a concentration range between 1 and 10 μM. For **Pc1-DMSO**, the intense Q-absorption band at 703 nm (log ε = 5.28) and a less intense Q-satellite band at 635 nm (log ε = 4.67) are characteristic for phthalocyanines in monomeric form. The Soret band exhibits two absorption maxima at 325 and 370 nm (Figure 3B and Table 1). For **Pc1-PBS**, an intense blue-shifted Q-absorption band appears at 660 nm with a reduced molar absorptivity of log ε = 4.73. The absorbance correlates linearly with concentration in the considered range according to the Lambert–Beer law. This suggests that predominately one species, namely dimers, forms in PBS that coexists with a fraction of monomers marked by the long wavelength maximum at 694 nm still present in the investigated concentration range. The blue-shifted Q-band (shifted to shorter wavelength) indicates the formation of H-type aggregates, in which the planar phthalocyanine rings are stacked face-to-face parallelly.^46,47^

**Table 1:** UV–Vis Absorption and Fluorescence Emission Maxima of **Pc1** in DMSO and in PBS in the Absence and Presence of Polymeric Carriers

	absorption							
	soret band		Q-bands				emission	
solvent/carrier	λ [nm]	log ε	λ_1_ [nm]	log ε	λ_2_ [nm]	log ε	λ_1_ [nm]	λ_2_ [nm]
DMSO	325/370	4.76/4.74	703	5.28	635	4.67	700–713	
PBS	318	4.79	694	4.73	660	4.75	700	
P188	318	4.88	696–702	5.01	660	5.02	699	717–719
P84	320	4.82	702	5.15	637	4.78	699	
F127	321	4.80	702	5.20	637–649	4.75	699	
RH40	321	4.77	702	5.33	635–637	4.68	699	
PVP	314	4.81	711	5.13	646–655	4.86	700	722

The fluorescence emission spectra of **Pc1-DMSO** and **Pc1-PBS** in the concentration range between 0.1 and 1.0 μM are shown in Figure 3D,E. The emission spectrum of **Pc1-DMSO** appears broad with maxima between 700 and 713 nm. This distribution of emission maxima may be associated with the presence of regioisomers of **Pc1** as discussed in Section 3.1. In a similar manner, the isolated regioisomers of tetra-α- substituted Mg-phthalocyanines were reported to exhibit slightly different fluorescence emission maxima.^48^ The linear correlation between fluorescence intensity and concentration in the investigated range (Figure S4A) underlines the presence of monomers as emitting species in DMSO. On the other hand, for **Pc1-PBS**, the fluorescence emission remains constant between 0.25 and 1 μM (Figure S4B). Most likely, this emission originates from a small persisting fraction of monomers whereas the H-aggregates, that are normally non-emissive, do not contribute to the fluorescence intensity in PBS.

Taken together, the TriEG tetra non-peripheral substitution of **Pc1** leads to a favorable shift of the absorption maximum toward the near infrared (NIR) wavelength region of light with λ_max_ around 700 nm and strongly enhances the water solubility compared to unsubstituted ZnPc.^8^ Even in the mM concentration range, in which the NMR spectra were recorded, no higher aggregates are observed. Nevertheless, the formation of H-type dimers already above a concentration of 0.1 μM leads to undesired losses of photodynamic activity supporting the need of suitable monomerizing carriers.

### 3.3 Encapsulation of **Pc1** into Polymeric Carrier Systems

#### 3.3.1 Selection of Polymeric Carriers

**Figure 4:**
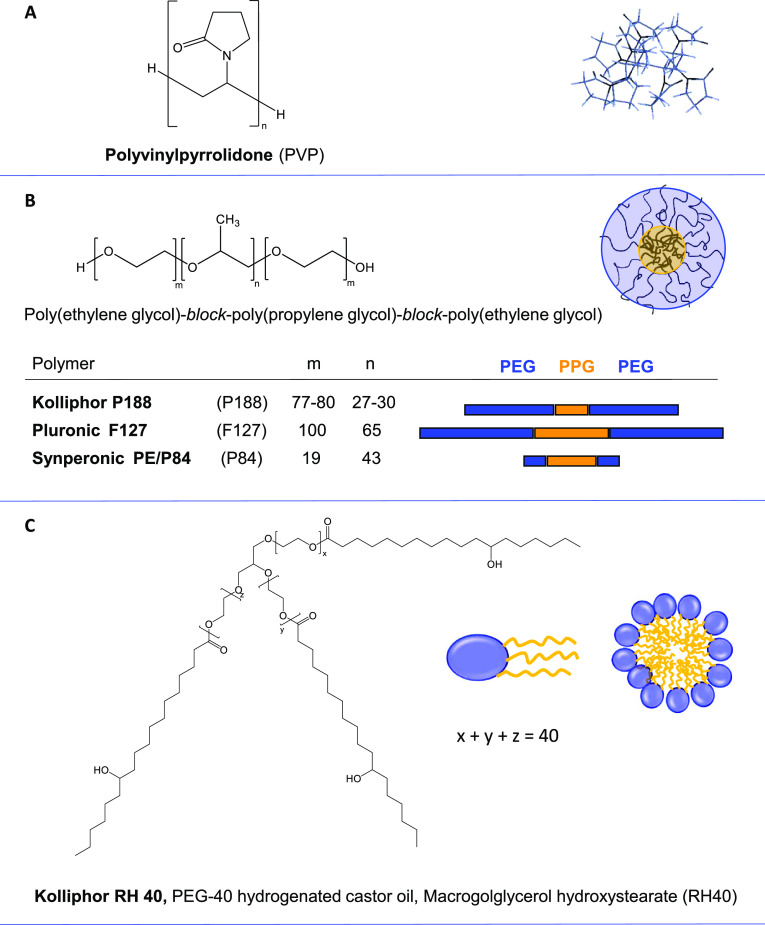
Structures of the polymeric carrier units. (A) Polyvinylpyrrolidone, PVP (B) Triblock copolymers, Kolliphor P188, Pluronic F127, and Synperonic P84. (C) PEG-40 hydrogenated castor oil (Kolliphor RH40). On the right, sketches of the PVP network, triblock copolymer micelles, and Kolliphor RH40 micelles.

For the comparative assessment of suitable carriers for the physical entrapment and monomerization of **Pc1**, it was mixed with different classes of polymeric drug delivery systems including PVP, PEG–PPG–PEG triblock copolymer micelles, and the micelle forming PEG ester of hydrogenated castor oil (Kolliphor RH40). The chemical structures and the supramolecular assemblies formed by the corresponding polymers are displayed in Figure 4. The rationale for this selection of polymeric carriers was (i) their good accessibility, (ii) ease of preparation, and (iii) broader range of coverage for hosting compounds with hydrophilic–lipophilic properties. PVP is a very versatile carrier material that forms a molecular network and is able to accommodate a relatively wide range of guest compounds.^49^ It has already gained approval among others in PDT as a drug delivery vehicle for the photosensitizer chlorin e6.^50^ The triblock copolymers PEG–PPG–PEG spontaneously form micelles in aqueous solutions above their critical micelle concentrations. Their properties can be tuned by varying the ratio and the total amount of PEG and PPG units, thus giving rise to different hydrophilicity–lipophilicity balance (HLB)-values and host-binding and dynamic properties.^30,51^ Overall, their structural similarity to the TriEG substituents of **Pc1** implies good compatibility of the triblock copolymer micelles for encapsulation. For the PEG–PPG–PEG triblock copolymers, different trade names combined with letters and numbers encoding the physical state (liquid, paste, or flake), molecular weight (MW), and PEG content are used as synonyms (Table S1), i.e., Pluronic, Kolliphor (from BASF) or Synperonic (from Croda). To simplify, they will be termed throughout the manuscript with their codes P188, F127, and P84, as listed in Figure 4B. Finally, to account for the relatively hydrophobic phthalocyanine macrocycle, the micelle forming PEG-ester of hydrogenated castor oil Kolliphor RH40 (abbreviated with RH40) was chosen that provides a large lipophilic environment with its lipid-like hydroxy-stearyl chains while offering a hydrophilic head group region formed by PEG units (for synonyms, see Table S2).^14^

#### 3.3.2 ^1^H NMR Spectroscopy

##### 3.3.2.1 ^1^H NMR Spectral Analysis of **Pc1** Resonances

**Figure 5:**
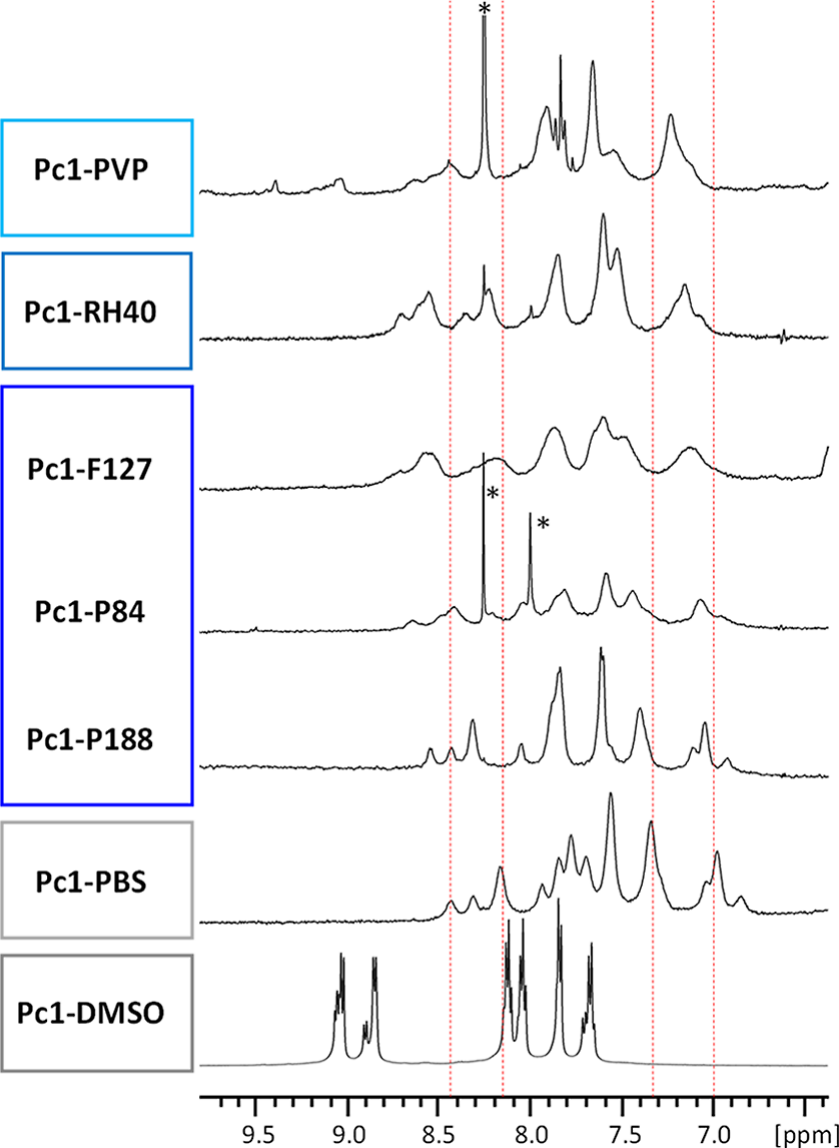
^1^H NMR spectral region showing the aromatic phthalocyanine resonances of **Pc1-DMSO**, **Pc1-PBS**, **Pc1-P188**, **Pc1-P84**, **Pc1-F127**, **Pc1-RH40,** and **Pc1-PVP**. The peaks marked with * are impurities from the polymer.

Inspection of the ^1^H NMR spectral aromatic region of **Pc1** after mixing with the different polymeric carrier systems in aqueous buffer solutions (Figure 5) allows estimation of its aggregation state and overall molecular surrounding. It becomes evident that P188 micelles are the least efficient for breaking up the dimeric structure of **Pc1** as there are only small differences in the spectral appearance of **Pc1-P188** compared to **Pc1-PBS**. For all other polymeric carrier systems, i.e., **Pc1-P84**, **Pc1-F127**, **Pc1-RH40**, and **Pc1-PVP**, a more or less pronounced downfield shift (to higher ppm values) of the resonances is observed with respect to the spectrum of **Pc1-PBS**. Since stacking of porphyrinic and phthalocyanine ring systems leads to ring-current-induced upfield shifts of the ^1^H NMR resonances from protons residing above or below the macrocyclic plane,^52^ the inversion of this effect reveals aggregate dissolution. However, the chemical shift of **Pc1** is not equal to that of the monomeric form present in DMSO. The difference may be caused either by the coexistence of monomers and dimers that are in fast exchange with each other on the NMR time scale or by the solvent effect.^53^ While DMSO forms a hydrophilic aprotic environment that is able to coordinate to the central Zn-atom of **Pc1**, the carrier systems rather provide hydrophobic surroundings. Apart from the chemical shift, the resonance line-broadening reveals information about the dynamic state of the molecule as discussed above (Section 3.2). Increased linewidths as observed for **Pc1** in the presence of all polymers but **Pc1-P188** compared to that for **Pc1-PBS** can be due to molecular exchange processes and decreased molecular mobility. The latter case is conceivable as the phthalocyanine inserts into micelles or molecular networks.

##### 3.3.2.2 ^1^H DOSY Spectra

**Figure 6:**
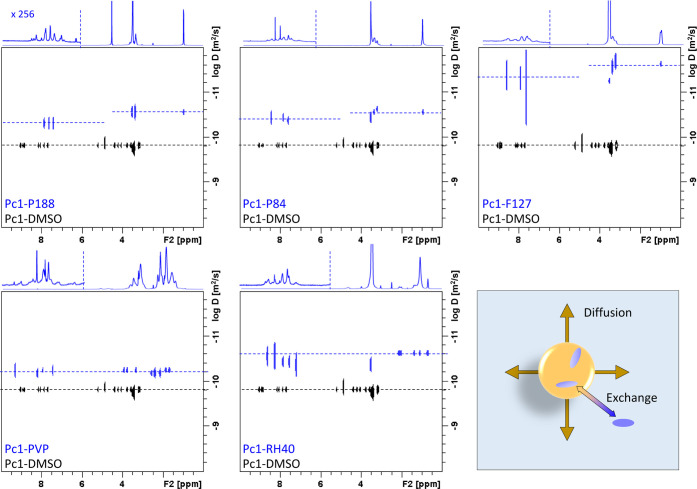
Overlay of ^1^H DOSY spectra of **Pc1-DMSO** (black) and **Pc1-P188**, **Pc1-P84**, **Pc1-F127** (blue, top row), **Pc1-PVP,** and **Pc1-RH40** (blue, bottom row). Sketch of dynamic processes reflected in the DOSY spectra (bottom right).

Association of guest molecules with macromolecules or supramolecular assemblies can be well studied by NMR diffusion measurements.^29,54^ In analogy to Figure 3A, an overlay of the DOSY spectra of **Pc1-DMSO** (shown in black) in the presence of the different polymeric carrier materials at a molar ratio of 1:10 (**Pc1**/polymer) in PBS (shown in blue) is shown in Figure 6. In the corresponding 1D ^1^H NMR projection spectra of the **Pc1**-polymer mixtures, the aromatic region is scaled up by a factor of 256 making the **Pc1** resonances visible besides the intense polymer peaks. From the DOSY plots, it becomes evident that the diffusion coefficient of **Pc1** is reduced to a value equal or similar to the diffusion coefficient of the polymer. This indicates polymer association of **Pc1**, meaning that phthalocyanine adopts the same diffusion properties as its supramolecular host so that the ensemble is freely diffusing as one particle (Figure 6, bottom right panel). If only one diffusing species exists in solution, the *D*-values of **Pc1** and the polymer appear in a single line in the DOSY spectrum as it is the case for **Pc1-PVP** and nearly also for **Pc1-RH40** (Figure 6 bottom row). The coexistence of two or more species that are in mutual exchange leads to the observation of diffusion values as weighted average.^55^ This is observed for **Pc1** in the presence of the triblock copolymer micelles, where the diffusion coefficient of **Pc1** is clearly reduced compared to **Pc1-DMSO**, but not in line with the polymer D-values (Figure 6 top row). Only a fraction of **Pc1** resides in the micellar environment whereas the other fraction still exists as dimers in the aqueous bulk solution. The gap between the *D*-values of the host and guest is indicative of the distribution equilibrium and can be exploited to estimate the association constants.^28,56^ While the chemical shift of the phthalocyanine resonances were hardly changed in **Pc1-P188** (Figure 5), the DOSY experiment suggests that the interaction between **Pc1** and P188 micelles takes place to some extent but without strong binding. The DOSY experiment has a high sensitivity for detecting this interaction, because there is a large difference in the *D*-values of the single **Pc1** molecule (or the dimer) with an MW of ∼1 kDa and the micelles consisting of multiple polymer molecules with an MW of 8.4 kDa (P188) per unit. In addition, the dynamics of triblock copolymer micelles offer a more fluid-like environment for encapsulated drug molecules allowing for exchange with the surrounding aqueous phase. The balance can be shifted toward the micellar compartment by changing the polymer composition, i.e., the PEG–PPG–PEG ratio, as well as the polymer–drug compatibility.

In summary, the DOSY spectra suggest that PVP and RH40 micelles are capable of binding **Pc1** with stronger repelling molecular exchange. Triblock copolymer micelles are capable of encapsulating **Pc1** but allow molecular exchange to different extents, the most pronounced for P188.

##### 3.3.2.3 ^1^H NMR Spectral Analysis of Polymer Resonances

**Figure 7:**
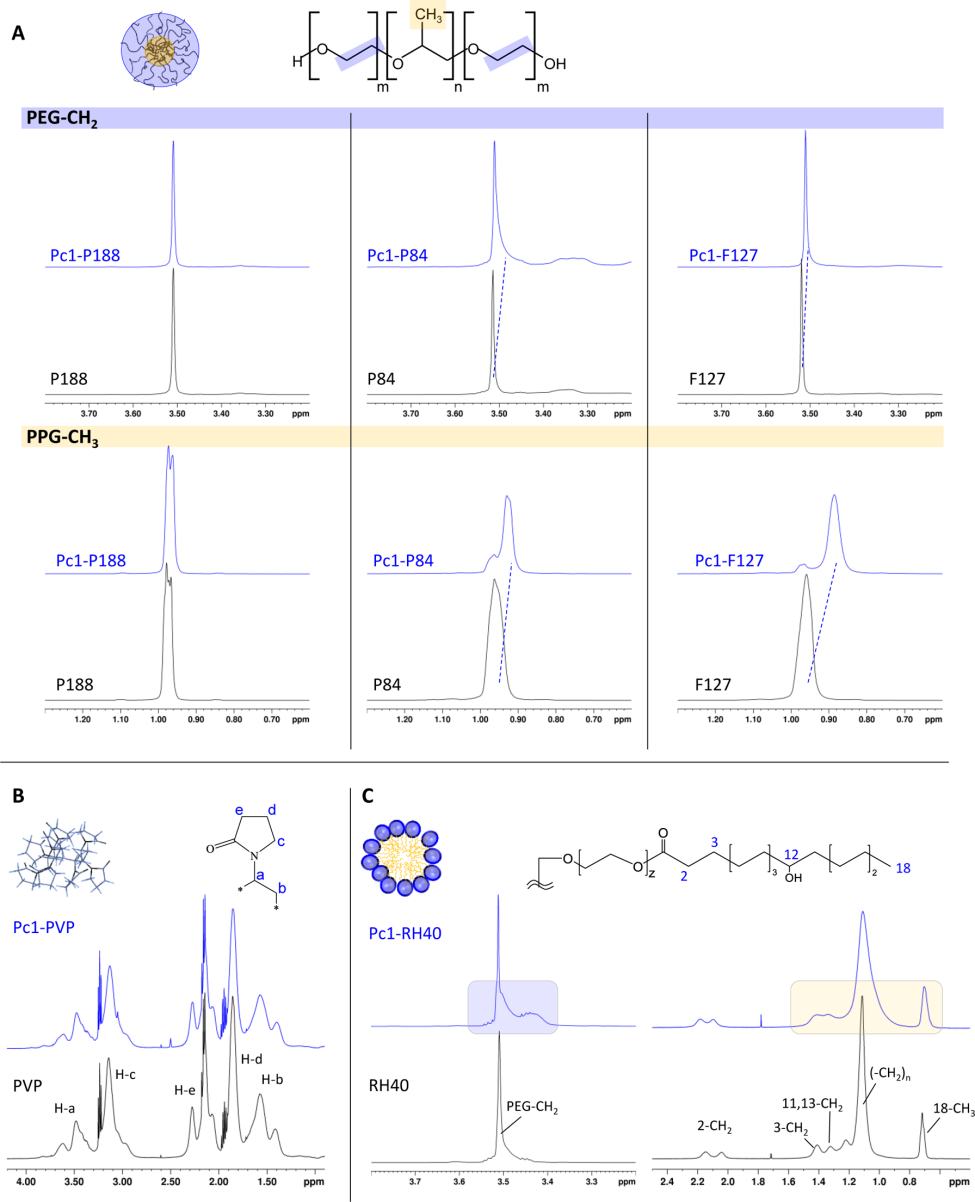
^1^H NMR spectra in the absence (black) and presence (blue) of **Pc1** of (A) triblock copolymer micelles composed of P188, P84, and F127. Displayed are the spectral regions of the PEG-CH_2_ resonance (top) and the PPG-CH_3_ resonance (bottom), (B) PVP, and (C) RH40 micelles.

To study encapsulation of **Pc1** in more detail, the ^1^H NMR resonances of the polymers were analyzed. In case of triblock copolymer micelles, both PEG and PPG chains give rise to two intense resonances, one around 3.5 ppm for the PEG-CH_2_ representing the micellar corona and the other at 0.9–1 ppm for the PPG-CH_3_ representing the micellar core (Figure 7A). Chemical shift perturbation of these polymer resonances induced by the ring current of the phthalocyanine macrocycle is indicative of **Pc1** proximity and allows mapping of the sites of intermolecular interactions with atomic resolution.^29,30,57,58^ While the P188 −CH_2_ and −CH_3_ resonances remained unperturbed in **Pc1-P188**, there was a clear upfield shift of the P84 and even more pronounced upfield shift of the F127 PPG-CH_3_ resonances in **Pc1-P84** and **Pc1-F127**, respectively (Figure 7A). On the contrary, the PEG-CH_2_ resonances experience only small shifts. Accordingly, the **Pc1** macrocycle resides in the micellar core with its TriEG chains most likely pointing toward the corona. Comparing the induced chemical shifts, it can be concluded that F127 containing the highest number of core-forming PPG units (Figure 4) is best suited among the three triblock copolymer types to accommodate **Pc1**.

As opposed to the shifted triblock copolymer resonances, no changes appeared for the PVP resonances in **Pc1-PVP** (Figure 7B). Nevertheless, binding of **Pc1** to the PVP network takes place based on the common diffusion properties as proved by the DOSY data of **Pc1-PVP** shown in Figure 6. The lack of ring current-induced shifts suggests that **Pc1** is mainly adsorbed to the PVP surface through interaction with the TriEG chains rather than through the interactions with the phthalocyanine macrocycle.

For **Pc1-RH40,** in turn, induced shifts and line broadening appeared for the hydroxy-stearic acid methylene [(-CH_2_)_n_] and terminal methyl (18-CH_3_) resonances indicating proximity of the phthalocyanine ring to the micellar core upon **Pc1** encapsulation. Other than for triblock copolymer micelles, however, for **Pc1-RH40**, an upfield shifted shoulder of the PEG-CH_2_ resonance emerged besides the unperturbed sharp PEG-CH_2_ peak most likely representing the interior and exterior regions of the micellar PEG-corona (Figure 7C). Accordingly, the induced shifts suggest that **Pc1** molecules reside in the micellar core reaching into the intersection toward the corona formed by the PEG units.

**Figure 8:**
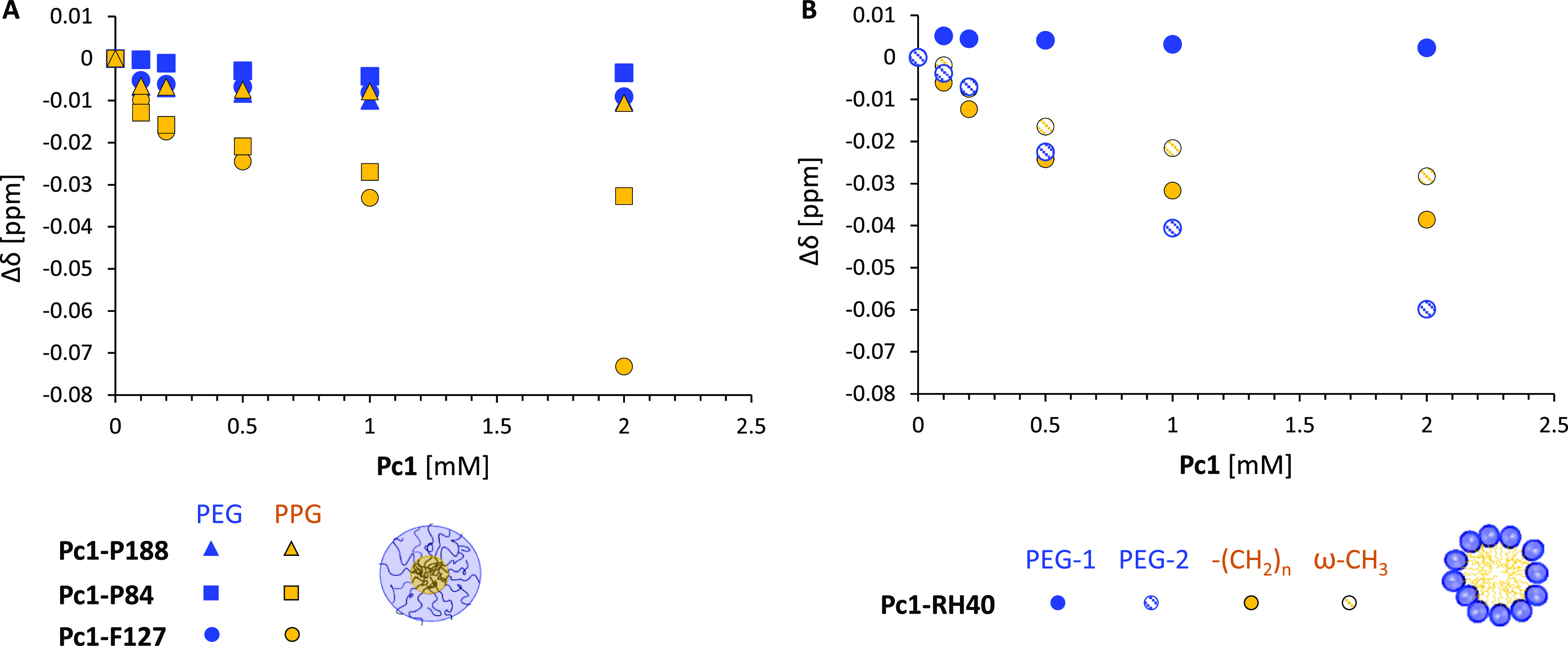
^1^H NMR chemical shift difference, Δδ, as the function of **Pc1** concentration for (A) PEG-CH_2_ and PPG-CH_3_ resonances of **Pc1-P188**, **Pc1-P84**, and **Pc1-F127** and for (B) PEG-CH_2_ and the stearic acid −(CH_2_)_*n*_ and ω-CH_3_ resonances of **Pc1-RH40**.

The magnitude of the induced upfield shift of the polymer resonances correlates with the distance between the polymer protons and the phthalocyanine macrocycle plane^52^ as well as with its concentration. In Figure 8, the chemical shift difference Δδ of the polymer resonances in the absence and presence of **Pc1** (eq 7) is plotted as function of **Pc1** concentration for the range of 0.1–2.0 mM at constant polymer concentration.
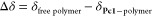
7

For the triblock copolymer micelles, the PEG-CH_2_ protons from the micellar corona experience no or very small shifts. On the other hand, saturation curves are obtained for the PPG-CH_3_ of **Pc1-P84** and **Pc1-F127** (Figure 8A). Likewise, for the stearic acid and the interior PEG protons of **Pc1-RH40**, the chemical shift approaches a limit with increasing **Pc1** concentration (Figure 8B). The continuous chemical shift changes with increasing DL suggest a homogeneous distribution with a fast exchange of the drug among the polymer chains within the micellar core. No noteworthy chemical shift changes appeared for the PVP resonances with increasing **Pc1** amounts in **Pc1-PVP** (Figure S5).

##### 3.3.2.4 ^1^H^1^H NOESY Spectra

**Figure 9:**
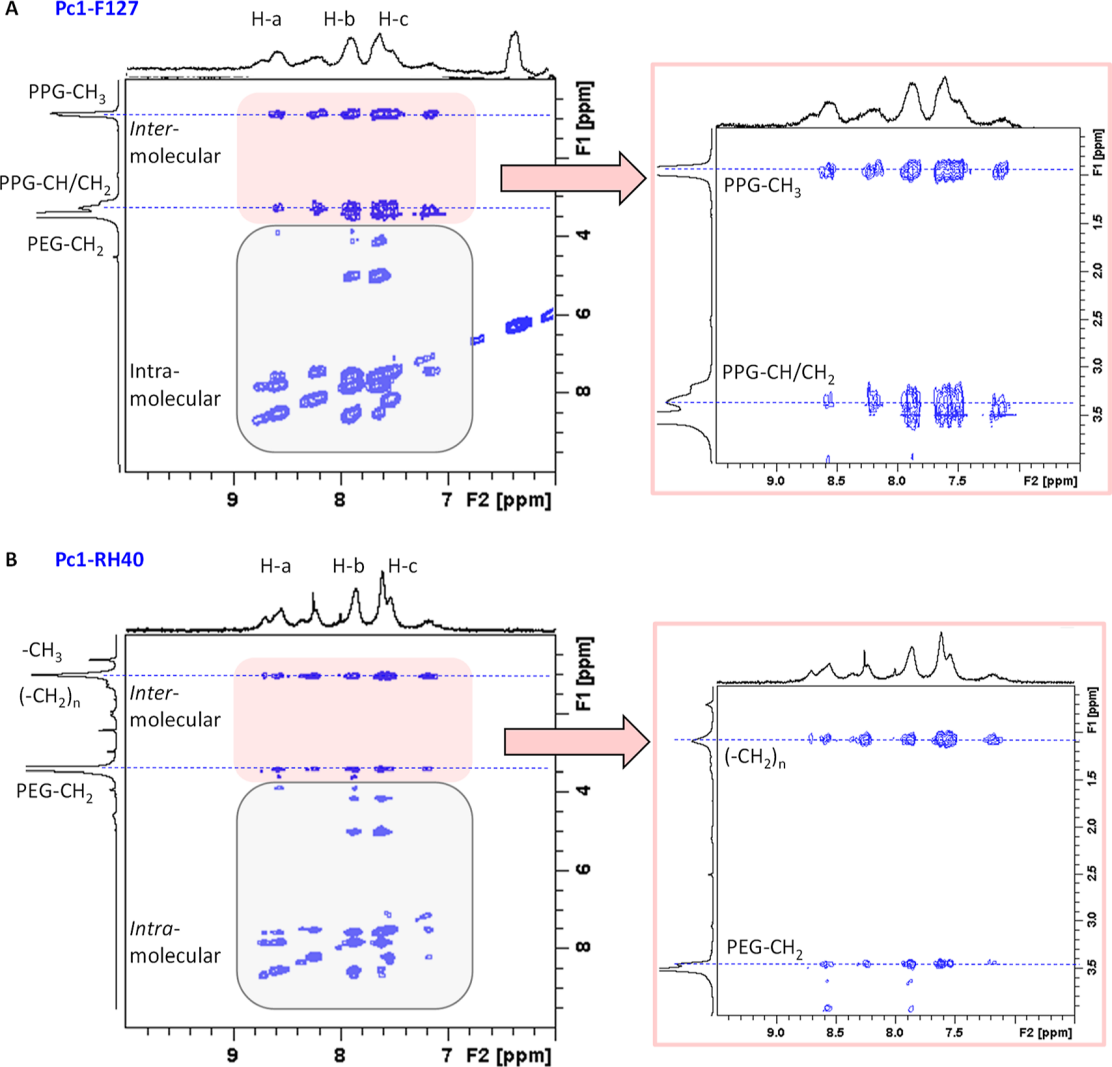
^1^H^1^H NOESY (*T*_m_ = 100 ms) spectra of (A) **Pc1-F127** and (B) **Pc1-RH40**. The red boxes highlight intermolecular NOEs between the aromatic protons of **Pc1** and the polymer resonances. Expansions are shown on the right.

The NOESY experiment allows detecting through-space interactions of nearby protons up to an internuclear distance of about 5 Å.^55,59^ In Figure 9, the ^1^H^1^H NOESY spectra of **Pc1-F127** (Figure 9A) and **Pc1-RH40** (Figure 9B) are displayed for the aromatic region along the F2-dimension, where the phthalocyanine resonances do not overlap with the polymer spectra. First, intense intramolecular NOEs appear among the aromatic protons of the phthalocyanine macrocycle and between the aromatic protons and the protons of the TriEG chains that are close to the ring system. Second, intermolecular NOEs appear between the aromatic phthalocyanine protons and the polymer protons. In **Pc1-F127**, the main contributions derive from interactions with the PPG-CH_3_ protons (peak at 0.95 ppm) and with the PPG-CH and −CH_2_ protons with peak positions close to the intense PEG-CH_2_ resonance (Figure 9A). In **Pc1-RH40**, NOE cross peaks appear between the aromatic phthalocyanine protons and the methylene (CH_2_)_*n*_ resonance of the stearic acid chain but not with the terminal −CH_3_ resonance. In addition, the aromatic protons give rise to NOE cross peaks with the PEG-CH_2_ protons in **Pc1-RH40** (Figure 9B). In the expansion of the NOESY spectrum, it becomes visible that the interaction is restricted to the PEG-protons contributing to the upfield shifted peak at around 4.4 ppm. Thus, the NOESY data support the conclusion that for **Pc1-RH40**, the phthalocyanine resides in the micellar core closer to the core–corona interface in RH40 micelles. For **Pc1-F127**, the phthalocyanine resides deeper in the PPG-forming core region of F127 micelles. Similar but less pronounced intermolecular NOEs were detected for **Pc1-P84** between the phthalocyanine and P84 protons whereas no intermolecular NOEs appeared for **Pc1-P188** (Figure S6A,B) supporting the assumption that P188 micelles do not efficiently bind **Pc1**. The importance of localization of Pc-based photosensitizers along the micellar cross section for the generation of reactive oxygen species has been previously pointed out, where the micellar core region was the most efficient microenvironment.^60^ NOESY spectra of **Pc1**-**PVP** were devoid of any intermolecular NOEs (Figure S6C) unless the NOE mixing time was prolonged from 100 to 500 ms making NOEs visible between the aromatic phthalocyanine and all PVP protons (Figure S6D). While this proves the proximity of **Pc1** to PVP, the distinct PVP protons directly interacting with **Pc1** protons cannot be distinguished as spin diffusion effects may prevail at long mixing times for large molecules.^59^

#### 3.3.3 UV–Vis Absorption and Fluorescence Spectroscopies

**Figure 10:**
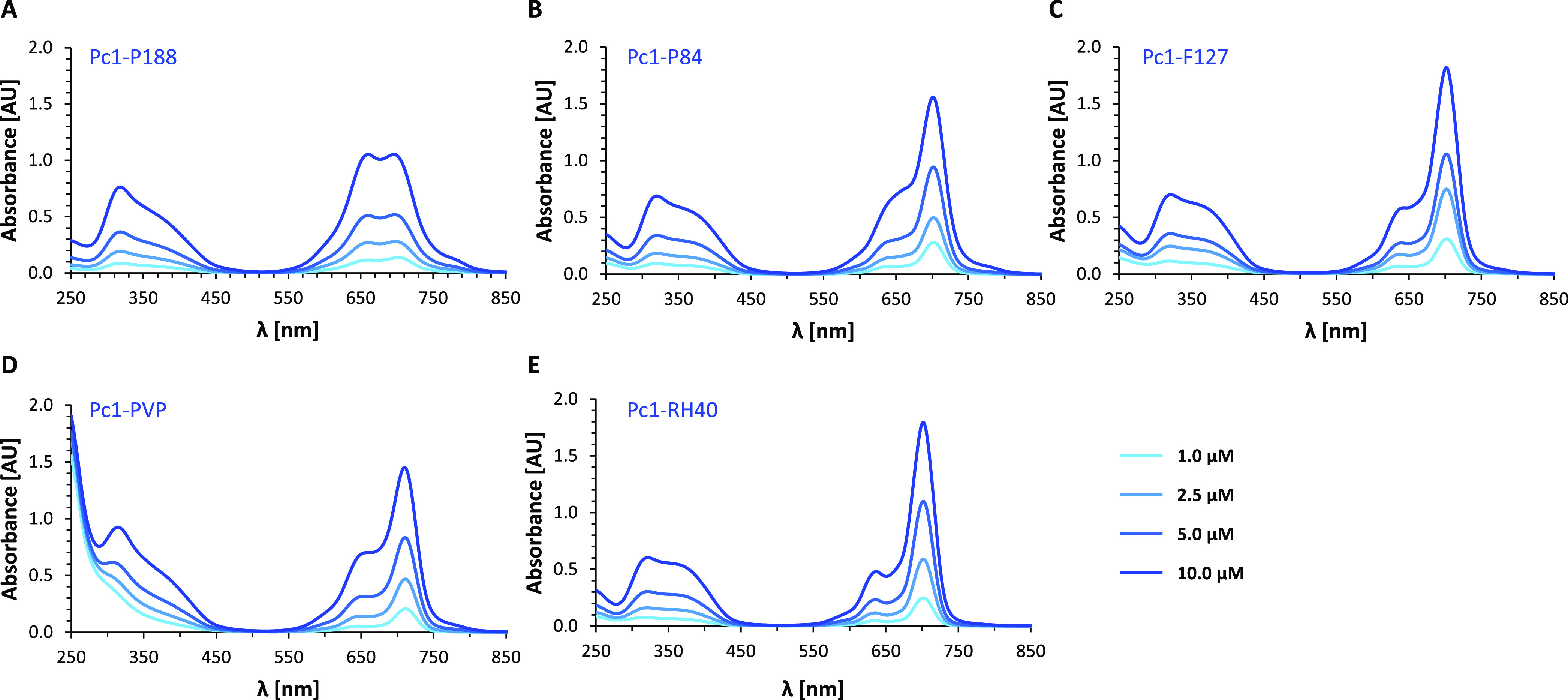
UV–vis spectra at **Pc1** concentrations of 1, 2.5, 5, and 10 μM. (A) **Pc1-P188**, (B) **Pc1-P84**, (C) **Pc1-F127**, (D) **Pc1-PVP**, and (E) **Pc1-RH40**.

**Figure 11:**
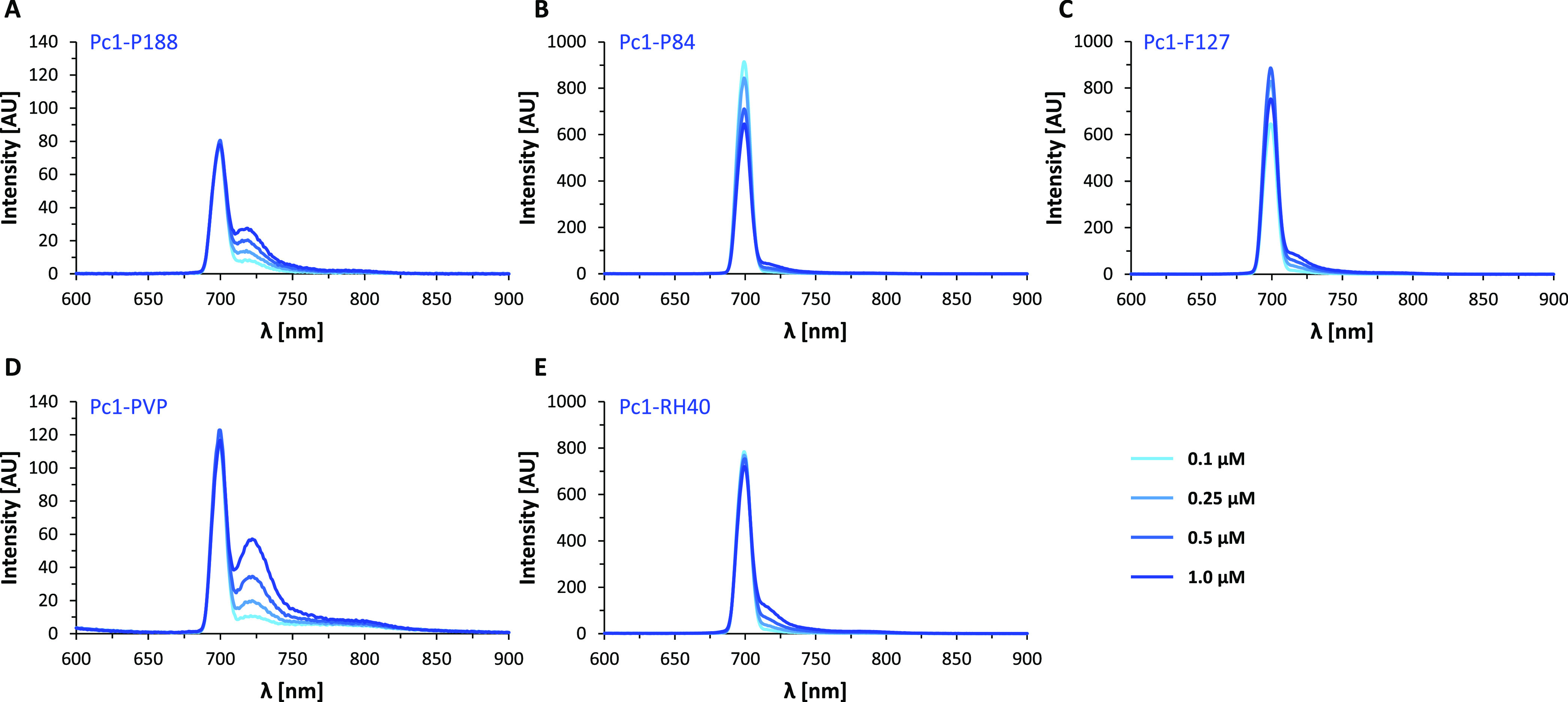
Fluorescence spectra at **Pc1** concentrations of 0.1, 0.25, 0.5, and 1 μM. (A) **Pc1-P188**, (B) **Pc1-P84**, (C) **Pc1-F127**, (D) **Pc1-PVP**, and (E) **Pc1-RH40**.

The UV–vis absorption spectra of **Pc1** combined with the five different polymers in aqueous PBS are displayed in Figure 10 for a **Pc1** concentration range of 1–10 μM and constant polymer concentration. The corresponding fluorescence emission spectra are given in Figure 11 for a **Pc1** concentration range of 0.1–1 μM. In **Pc1-P188**, H-type aggregates persist as indicated by the strong absorption of the blue-shifted band at 660 nm (Figure 10A), which is similar to the absorption spectra of **Pc1-PBS** (Figure 3C). This is in agreement with the NMR results of insufficient interactions of P188 with **Pc1** for aggregate dissolution. In analogy to **Pc1-PBS**, low-intensity fluorescence emission of **Pc1-P188** (Figure 11A) is most likely due to the small monomer fraction of **Pc1** (λ_1 max_ = 699 nm). Noteworthily, an additional low-intensity fluorescence emission maximum appears at a longer wavelength (λ_2 max_ = 718 nm) that correlates with **Pc1** concentration (Figure S4D). The absorption spectra of **Pc1-P84** and **Pc1-F127** exhibit increasing monomeric character with a reduction in the absorbance at 660 nm and an increase at 702 nm with molar extinction coefficients of log ε 5.15 and 5.20, respectively (Figure 10B,C and Table 1). Both triblock copolymer micelles lead to a significant increase in the fluorescence emission intensity values of **Pc1-P84** and **Pc1-F127** to values more than ten-fold higher compared to those of **Pc1-PBS** or **Pc1-P188** (Figure 11A–C). A very similar result as for **Pc1-F127** is obtained for **Pc1-RH40**. The absorption spectrum of **Pc1-RH40** resembles that of monomeric **Pc1-DMSO** and exhibits the largest molar absorption coefficient among the different media with log ε of 5.33 (λ _max_ = 702 nm) and intense fluorescence emission (λ _max_ = 699 nm). In **Pc1-PVP**, the absorption maximum of the Q-band is redshifted to 711 nm. In the fluorescence spectra, an additional emission band appears at longer wavelengths (λ_max_ = 725 nm) of which the fluorescence intensity correlates linearly with **Pc1** concentration (Figure S4H). This indicates that at least, in part, J-aggregates may form upon association with the PVP surface. J-aggregates are rather rarely formed by phthalocyanines and are characterized by redshifted Q-absorption bands and–as opposed to H-aggregates–by intense fluorescence.^46,61^ Since the total intensity of the fluorescence emission band is still rather low, there is most likely a mixture of mainly H-aggregates (non-emissive), small fractions of monomers (700 nm), and J-aggregates (722 nm) present in **Pc1-PVP**. Similarly, the presence of mixed monomers/dimers as well as mixed J- and H-type aggregates was suggested for zinc and aluminum phthalocyanines, respectively, when associated with PVP based on their absorption spectra.^23,62^

#### 3.3.4 Encapsulation Efficiency and Drug Loading

To further assess the degree of **Pc1**–polymer interaction, the EE and the DL were determined for each of the carriers at a molar ratio of 1:10 (**Pc1**:polymer) in PBS as applied elsewhere in this study. For this, the non-entrapped fraction of **Pc1** was quantified by UV–vis absorption spectroscopy (Figure S7) after size-selective separation from the large **Pc1**–carrier assemblies via ultrafiltration. The results are summarized in Table 2.

**Table 2:** EE and DL of **Pc1** in the Polymeric Carriers at a Molar Ratio of 1:10 (**Pc1**/Polymer) in PBS

carrier	P188	P84	F127	RH40	PVP (10 kDa)	PVP (40 kDa)^a^
EE %	2.82	50.73	67.18	88.42	64.75	63.03
DL %	0.04	1.46	0.65	4.16	0.79	0.19

a For validation, EE and DL were also determined for PVP with MW of 40 kDa.

In accordance with the NMR and optical data, the EE of **Pc1** was very low for P188 indicating weak interactions. It was significantly higher for the triblock copolymer micelles formed by P84 and F127 with EE values of 50.7 and 67.2%, respectively. Moreover, the graph shown in Figure 8A implies that the maximum **Pc1** encapsulation is nearly reached at a molar ratio of 1:10 for P84 while it still increases for F127 at higher drug-to-polymer ratios. The most efficient **Pc1** encapsulation was found for RH40 micelles with an EE of 88.4% and a DL of 4.16%. Similar to F127, Figure 8B indicates that the DL can be further increased for RH40 at higher drug-to-polymer ratios. The **Pc1-PVP** system exhibited a relatively high EE with 64.8% suggesting quite strong interactions, which is in agreement with the NMR data (Figures 6 and S6D). Since **Pc1-PVP** was just above the MWCO (10 kDa) of the filter membrane, for validation, EE and DL were also determined for PVP with an MW of 40 kDa that yielded a similar EE value (63%).

#### 3.3.5 Proposed Binding Schemes of Pc1 to Polymers

**Figure 12:**
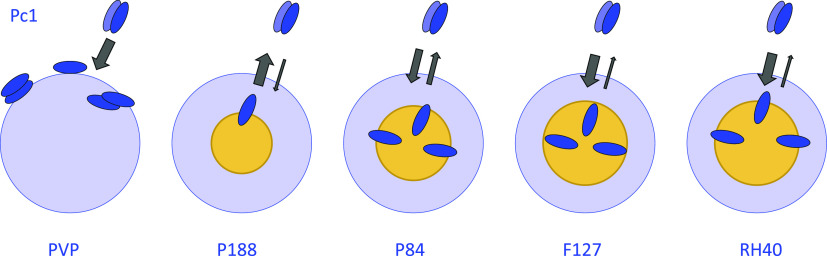
Proposed binding schemes of **Pc1** to the different polymeric carriers. Exchange processes are indicated by arrows, and their equilibrium is represented by the arrow width.

In summary, the five different polymeric carrier materials investigated here exhibit different binding schemes and encapsulation capacities for **Pc1**. This is delineated schematically in Figure 12. Binding strength and capability for monomerizing **Pc1** decreas for the triblock copolymer micelles in the order **Pc1-F127** > **Pc1-P84** > **Pc1-P188** and correlates with the number of the micellar core-forming PPG units. For **Pc1-RH40**, the phthalocyanine molecules reside at the interface between micellar core and corona, and the equilibrium is mostly shifted to the micellar environment due to the relatively strong binding. Likewise, for **Pc1-PVP**, the main fraction of **Pc1** is associated with PVP, but here, **Pc1** molecules are rather surface-attached and exist as monomer/(H- and J-type)dimer mixtures.

#### 3.3.6 Singlet Oxygen Quantum Yield

The photodynamic effect mainly relies on the formation of singlet oxygen (^1^O_2_) via energy transfer from the triplet excited state of the photosensitizer to molecular oxygen (Type II reaction), ^1^O_2_ being the genuine cytotoxic agent.^63^ Hence, the photo-generated amount of ^1^O_2_ is a key feature for predicting the PDT efficiency of a photosensitizing system. The quantum yield of ^1^O_2_ generation (Φ_Δ_) by **Pc1** was therefore determined for the different formulations in PBS. To benefit from the extended ^1^O_2_ lifetime in D_2_O compared to H_2_O^64^ thereby facilitating ^1^O_2_ detection, PBS-D_2_O was used. The method was based on direct detection of ^1^O_2_ phosphorescence using MB as the reference. The data are summarized in Table 3. The **Pc1-F127**, **Pc1-RH40**, and **Pc1-PVP** systems exhibited efficient ^1^O_2_ generation with Φ_Δ_ values of 0.24, 0.31, and 0.41, respectively, when compared to literature values of phthalocyanines obtained in aqueous solutions,^65^ while no ^1^O_2_ formation was detected for **Pc1-PBS**, **Pc1-P188,** and **Pc1-P84**. These results can be correlated with the aggregation state of the phthalocyanine in these different carriers, since aggregation quenches the formation of ^1^O_2_^66^ as it is the case for **Pc1-PBS** and **Pc1-P188**. Stronger binding of monomeric **Pc1** to the carrier and suppression of exchange with the surrounding aqueous medium (Figure 6) seem to correlate with ^1^O_2_ formation and promote higher Φ_Δ._values.

**Table 3:** Singlet Oxygen Quantum Yield (Φ_Δ_) of **Pc1** Alone and Encapsulated into the Carriers in PBS-D_2_O

system	Pc1	Pc1-P188	Pc1-P84	Pc1-F127	Pc1-RH40	Pc1-PVP
Φ_Δ_	nd^a^	nd^a^	nd^a^	0.24 ± 0.03	0.31 ± 0.05	0.41 ± 0.03

a No ^1^O_2_ emission detected.

### 3.4 FLIM In Vitro Imaging of **Pc1**–Polymer Mixtures

**Figure 13:**
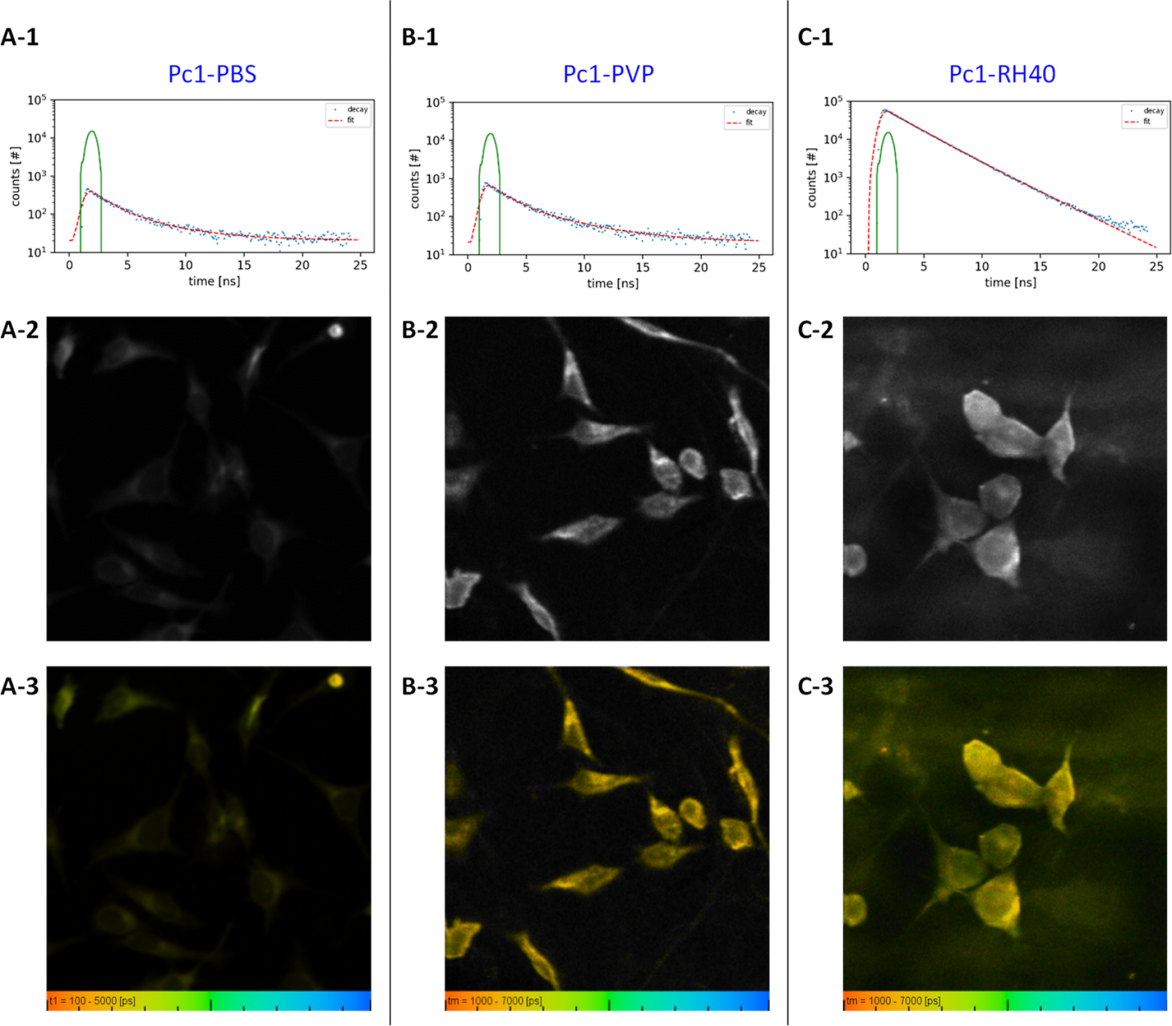
Fluorescence lifetime decay curves of incubation solutions (top row), fluorescence intensity images (middle row), and fluorescence lifetime images (bottom row) of HeLa cells incubated with (A) **Pc1-PBS**, (B) **Pc1-PVP**, and (C) **Pc1-RH40**.

Time-resolved fluorescence measurements allow the determination of fluorescence lifetimes of fluorophores, and their decay dynamics are sensitive to molecular interactions. Combined with spatial resolution through microscopy, FLIM can thus offer Supporting Information about the aggregation state and direct molecular surrounding of the phthalocyanine in complex environments like cells.^67,68^ To study the fate of **Pc1** in the cellular environment, HeLa cells were incubated for 24 h in the dark with **Pc1-PBS**, **Pc1-PVP**, and **Pc1-RH40**. The fluorescence decay curves of the corresponding incubation solutions are displayed in Figure 13A(1)–C(1) and the fluorescence lifetimes are summarized in Table 4. The decay curves of **Pc1** appeared biexponential in each of the media. In **Pc1-DMSO**, where monomers exist, the main component decays at τ_f_ = 2.2 ns. As outlined above, the steady-state fluorescence emission of **Pc1-PBS** and **Pc1-PVP** is mainly attributed to the residual fraction of monomers and possibly J-aggregates (in case of **Pc1-PVP**) besides a larger fraction of non-emissive H-aggregates (see Sections 3.2 and 3.3.3). Therefore, the fluorescence decay curves exhibit low initial count rates and only derive from the low monomer/J-aggregate fractions. The long-lived components (τ_f_ of 4.4, 4.7 ns) most likely arise from the monomers present in solution whereas the second short-lived component (τ_f_ of 1.5, 1.4 ns) reflects the involvement of aggregates/dimers that are prevalent in these media. According to detailed fluorescence lifetime studies reported on sulfonated aluminum phthalocyanine (AlPc) monomers and dimers, non-fluorescent ground state dimers can quench the monomer fluorescence in a concentration-dependent manner.^68−71^ Thus, the shortened lifetimes of **Pc1-PBS** and **Pc1-PVP** can be a result of fluorescence quenching of monomers via energy transfer to the coexistent H-type dimers. At least in **Pc1-PVP**, the short component may also be attributed to J-type aggregates present based on the UV–vis and steady-state fluorescence emission data. However, since the single lifetime values and their corresponding contributions are very similar for **Pc1-PBS** and **Pc1-PVP** (Table 4), it is conceivable that the same underlying processes determine the decay dynamics in these systems. For **Pc1-RH40**, decay times of 2.2 and 2.9 ns (59%) but no short component typical for aggregates like in **Pc1-PBS** or **Pc1-PVP** were observed. The two decay times of **Pc1-RH40** may reflect the different surroundings at the RH40–micellar interfaces corresponding to the **Pc1** distribution between the micellar core and the core/corona transition area as indicated by the NMR data (see 3.3.2). Similarly, liposomes encapsulating AlPc derivatives have been considered a microheterogeneous environment possibly giving rise to different decay times.^70^ Finally, the solvent effects can explain the differences in the lifetimes of monomers in the various media.^67^ With decreasing polarity of the surrounding medium, a shortening of the fluorescence lifetime is typically observed, which explains the longer τ_f_ values for **Pc1-PBS** and **Pc1-PVP** compared to **Pc1-DMSO** and the hydrophobic environment of the micellar cores in **Pc1-RH40**.

**Table 4:** Fluorescence Lifetimes of **Pc1-DMSO**, **Pc1-PBS**, **Pc1-PVP**, and **Pc1-RH40** from Biexponential Fitting Giving Rise to the Lifetimes τ_1_ and τ_2_ and the Corresponding Fractions A_1_ and A_2_ (See 2.3.4)

	fluorescence lifetime							
	solution				HeLa cells (24 h)			
solvent/carrier	τ_1_ [ns]	A1 [%]	τ_2_ [ns]	A2 [%]	τ_1_ [ns]	A1 [%]	τ_2_ [ns]	A2 [%]
**Pc1-DMSO**	2.2	85	3.2	15				
**Pc1-PBS**	1.5	73	4.4	27	1.6	84	5.6	16
**Pc1-PVP**	1.4	71	4.7	29	1.7	81	6.1	19
**Pc1-RH40**	1.9	41	2.9	59	2.2	90	9.6	10

Following the acquisition of fluorescence intensity images (Figure 13A(2)–C(2)), FLIM images of the cells were generated displaying the color-encoded weighted average fluorescence lifetime for each pixel (Figure 13A(3)–C(3)). The single fluorescence lifetime values obtained from biexponential fitting (averaged over all pixels) are listed in Table 4. According to the fluorescence intensity images, intracellular accumulation of **Pc1** was very low or only existent as non-fluorescing H-aggregates when applied as **Pc1-PBS** without carriers (Figure 13A(2)).

On the contrary, incubation with **Pc1**-**PVP** and with **Pc1**-**RH40** yielded relatively intense fluorescence in the peripheral intracellular areas around the nucleus. Both carriers are thus capable to deliver **Pc1** into the cell. The main cell uptake routes for nanoparticles are not only via the endocytic pathway but also via passive diffusion.^72,73^ Intracellular redistribution and interactions with cellular components, like proteins, will affect the fluorescence decay dynamics in the cellular environment. When applied as **Pc1-PVP**, **Pc1** exhibited a similar fluorescence lifetime distribution as in solution, which indicates that the phthalocyanine may remain associated with the PVP carrier inside the cells. Cell incubation with **Pc1**-**RH40** leads to a predominant component (90%) with a decay time of 2.2 ns equal to the decay time of **Pc1-DMSO** monomers. Compared to the τ_f_ values in solution, this suggests redistribution to a mostly uniform intracellular microenvironment of **Pc1** when delivered as **Pc1-RH40**. Intracellular redistribution of unsubstituted ZnPc applied with a similar carrier system (Cremophor EL, synonyms Kolliphor EL, and PEG-35 castor oil, Table S2) has been reported to take place to mitochondrial, lysosomal, and endoplasmic reticulum membranes.^74^ Similarly, **Pc1** delivered as **Pc1-RH40** may be mainly localized in membranes of various cell organelles. In summary, both, PVP and RH40 micelles are able to shuttle **Pc1** into the cells maintaining their fluorescence properties while **Pc1-RH40** results in more homogenous and longer intracellular fluorescence lifetimes and thus most likely resulting in higher photosensitizing efficiency than **Pc1-PVP**. The impact of triblock copolymer micelle mediated cell internalization of **Pc1** remains to be studied. It is known that PEG–PPG–PEG micelles are taken up by cells^75^ including HeLa cells.^26,76,77^ Based on the weak interactions observed here between **Pc1** and P188, the results for P188 are expected to be similar to those obtained for pure PBS (Figure 13A). However, for **Pc1-P84** and **Pc1-F127,** the relatively high intermolecular binding affinity is expected to yield sufficient intracellular **Pc1** as found for the **Pc1-RH40** and **Pc1-PVP** systems. Nevertheless, the carrier-specific total amount of **Pc1** taken up by the cells will be another important parameter to be determined in future studies.

## 4 Conclusions

In the current study, the tetra-substituted Zn phthalocyanine **Pc1** bearing TriEG chains in the non-peripheral positions was investigated with respect to its photophysical and aggregation properties in DMSO and in aqueous solutions in the absence and presence of five different polymeric drug delivery systems. **Pc1** exists as mixture of regioisomers in monomeric form in DMSO but forms dimers in water lowering its light absorption, fluorescence emission, and thus also the prospect of high PDT efficiency. Incorporation of **Pc1** into micelles consisting of either the triblock copolymer Kolliphor F127 (**Pc1-F127**) or the self-emulsifying drug delivery system Kolliphor RH40 (**Pc1-RH40**) was best suited to obtain stable **Pc1** monomers with significantly enhanced fluorescence intensity compared to **Pc1-DMSO** or **Pc1-PBS**. Three of the investigated carrier systems, **Pc1-F127**, **Pc1-RH40**, and **Pc1-PVP**, exhibited highest EE and efficient ^1^O_2_ quantum yields. Based on NMR chemical shift and NOESY data, a model for carrier-specific localization of the phthalocyanine at the polymer interface could be deduced. This, in turn, determines the likelihood of potential exchange processes or premature drug release. Localization of **Pc1** in the hydrophobic core of F127 and RH40 micelles could stabilize the monomeric form. Strong binding was also observed to PVP as a carrier while the monomerization of **Pc1** was less efficient in **Pc1-PVP**. However, both carrier types, **Pc1-RH40** micelles and **Pc1-PVP**, were capable to shuttle **Pc1** into HeLa cells while maintaining the phthalocyanines’ fluorescence properties, as shown by FLIM results.

The comparative study underlines the importance of fine-tuning carrier properties for matching the requirements to accommodate a certain molecular structure with respect to size and polarity. With this approach, simple and cost-effective polymeric carrier systems for physical entrapment are capable of increasing the drug efficiency significantly, and their usage is suitable for upscaling. In general, the chemical synthetic design of new active pharmaceutical drugs with enhanced efficiency and reduced side effects is nowadays mostly accompanied by the inevitable combination with suitable carrier systems as has been shown for similar metal complex-based compounds like cisplatin drugs^78^ or their ruthenium-based alternatives.^79^ For phthalocyanine-based photosensitizers, proof of the monomeric state and, in particular, the fluorescence properties in their final formulation and inside cells allow predicting their potential as efficient PDT drugs. To this end, the presented data have shown that the combination of NMR and optical spectroscopy for in vitro studies can provide a powerful predictive tool in the development of promising phthalocyanine formulations for PDT.

## Supporting Information Available

2D ^1^H^1^H COSY, 2D ^1^H^13^C HSQC, and 2D ^1^H^1^H NOESY spectra of **Pc1** in DMSO-*d*_6_; plots of fluorescence emission as function of **Pc1** concentration for **Pc1-DMSO**, **Pc1-PBS**, **Pc1-P188**, **Pc1-P84**, **Pc1-F127**, **Pc1-PVP**, and **Pc1-RH40**; ^1^H NMR spectra of **Pc1-PVP** in PBS at increasing **Pc1** concentrations; 2D ^1^H^1^H NOESY spectra of **Pc1-P188**, **Pc1-P84**, and **Pc1-PVP** at mixing times of 100 ms and 500 ms; UV–vis absorption spectra of filtrates obtained after ultracentrifugation of **Pc1**–polymer mixtures and of **Pc1-PBS** (1 mM); list of selected Triblock copolymers (PEG–PPG–PEG) and their synonyms; and list of selected PEGylated castor oils and their synonyms (PDF)

## ABBREVIATIONS

AlPc: Aluminum phthalocyanine
DL: Drug loading
DMSO: Dimethyl sulfoxide
DOSY: Diffusion-ordered spectroscopy
EE: Encapsulation efficiency
EG: Ethylene glycol
F127: Pluronic F-127
FLIM: Fluorescence lifetime imaging microscopy
HLB: Hydrophilicity–lipophilicity balance
MB: Methylene Blue
MEM: Minimum essential medium
MW: Molecular weight
MWCO: Molecular weight cutoff
NIR: Near infrared
NMR: Nuclear magnetic resonance
NOESY: Nuclear Overhauser enhancement spectroscopy
P188: Kolliphor P188
P84: Synperonic PE/P84
PBS: Phosphate-buffered saline
Pc1: 1, 8(11), 15(18), 22(25)-tetrakis-(2-(2-(2-methoxyethoxy)ethoxy) ethoxy)-Zn(II) phthalocyanine
PDT: Photodynamic therapy
PEG: Polyethylene glycol
PFA: Paraformaldehyde
PPG: Polypropylene glycol
PVP: Polyvinylpyrrolidone
RH40: Kolliphor RH 40
TriEG: Triethylene glycol
ZnPc: Zn(II) phthalocyanine
