# Supplementary material for: Comparing PVP and Polymeric Micellar Formulations of a PEGylated Photosensitizing Phthalocyanine by NMR and Optical Techniques
Source: Mol Pharm. 2023 Jul 26;20(8):4165–83. doi: 10.1021/acs.molpharmaceut.3c00306 (PMC10410667; doi:10.1021/acs.molpharmaceut.3c00306)
Supplement: Supplementary file 1 — mp3c00306_si_001.pdf [file mp3c00306_si_001.pdf]

SUPPORTING INFORMATION

Comparing PVP and Polymeric Micellar Formulations of a PEGylated Photosensitizing Phthalocyanine by NMR and Optical Techniques

Lea P. Gergely[a], Çiğdem Yüceel[b], Ümit İşci[c,d], Florentin S. Spadin[e], Lukas Schneider[f], Bernhard Spingler[f], Martin Frenz[e], Fabienne Dumoulin[g]*, and Martina Vermathen[a]*

[a] Department of Chemistry, Biochemistry and Pharmaceutical Sciences, University of Bern, 3012 Bern, Switzerland.

[b] Department of Chemical Engineering, Gebze Technical University, Gebze, 41400 Kocaeli, Türkiye.

[c] Department of Chemistry, Gebze Technical University, Gebze, 41400 Kocaeli, Türkiye.

[d] Marmara University, Faculty of Technology, Department of Metallurgical & Materials Engineering, 34722 Istanbul, Türkiye.

[e] Institute of Applied Physics, University of Bern, 3012 Bern, Switzerland.

[f] Department of Chemistry, University of Zurich, 8057 Zurich, Switzerland.

[g] Acıbadem Mehmet Ali Aydınlar University, Faculty of Engineering and Natural Sciences, Biomedical Engineering Department, Ataşehir, Istanbul, Türkiye.

Content

Figure S1: $^1H^1H$ COSY spectra of **Pc1** in DMSO-d6.

Figure S2: $^1H^{13}C$ HSQC spectrum (with multiplicity editing; red: CH, CH_3, blue: CH_2) of **Pc1** in DMSO-d_6.

Figure S3: $^1H^1H$ NOESY spectrum of **Pc1** in DMSO-d_6 (mixing time T_m = 500 ms).

Figure S4: Fluorescence emission at λ_{max} (em) as function of **Pc1** concentration for **Pc1-DMSO, Pc1-PBS, Pc1-P188, Pc1-P84, Pc1-F127, Pc1-PVP, and Pc1-RH40**; λexc = 350 nm.

Figure S5: ^1H NMR spectra of **Pc1-PVP** (10 mM PVP) in PBS at increasing concentrations of **Pc1**.

Figure S6: $^1H^1H$ NOESY spectra of **Pc1-P188**, **Pc1-P84**, and **Pc1-PVP** at a mixing time T_m of 100 ms and 500 ms.

Figure S7: UV-vis absorption spectra of filtrates obtained after ultracentrifugation of **Pc1**-polymer mixtures and of **Pc1-PBS** (1 mM).

Table S1: List of selected Triblock-copolymers (PEG-PPG-PEG) and their synonyms.

Table S2: List of selected PEGylated Castor oils and their synonyms.

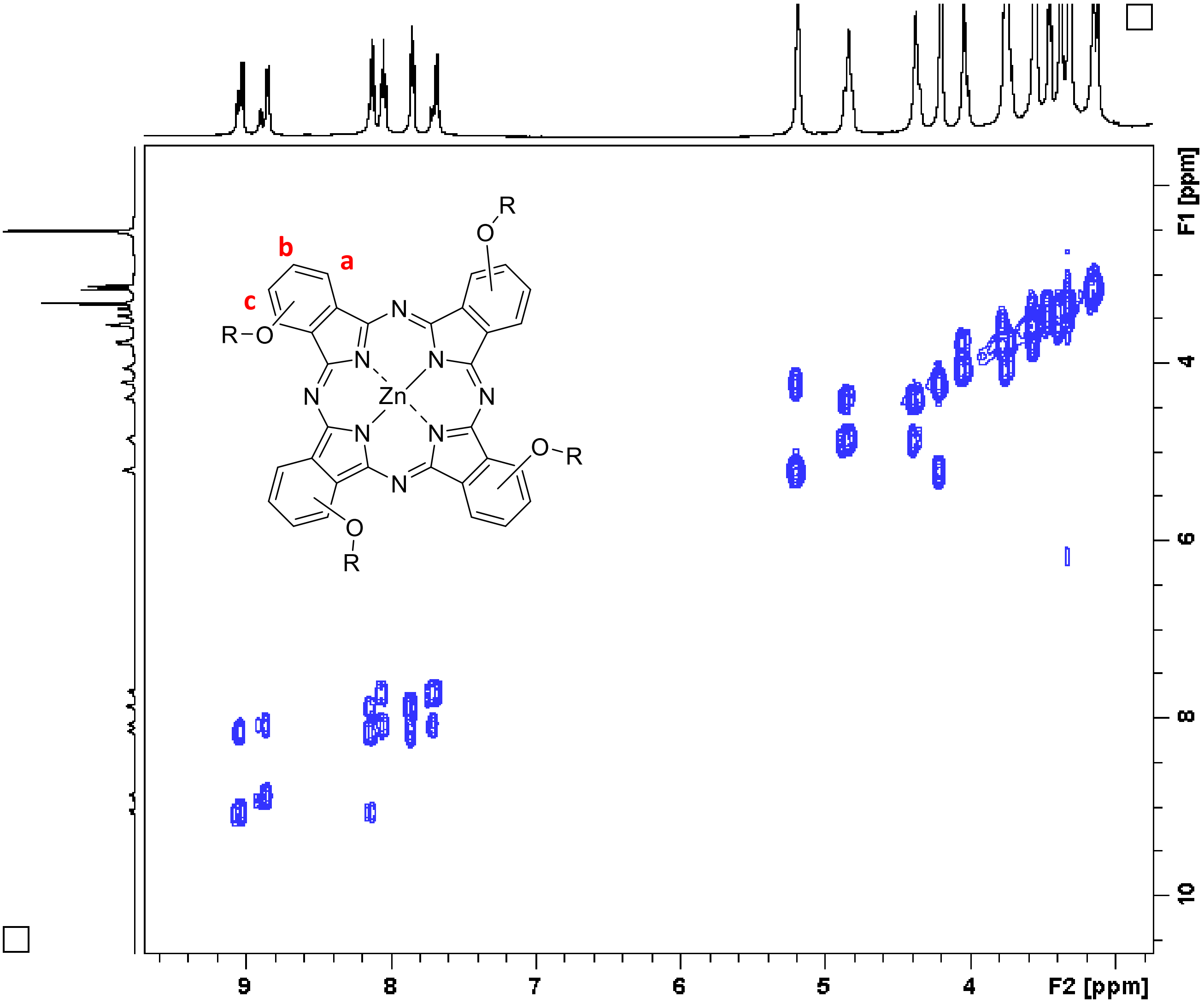

Figure S1 A: $^1H^1H$ COSY spectrum of **Pc1** in DMSO-d_6.

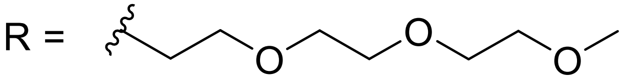

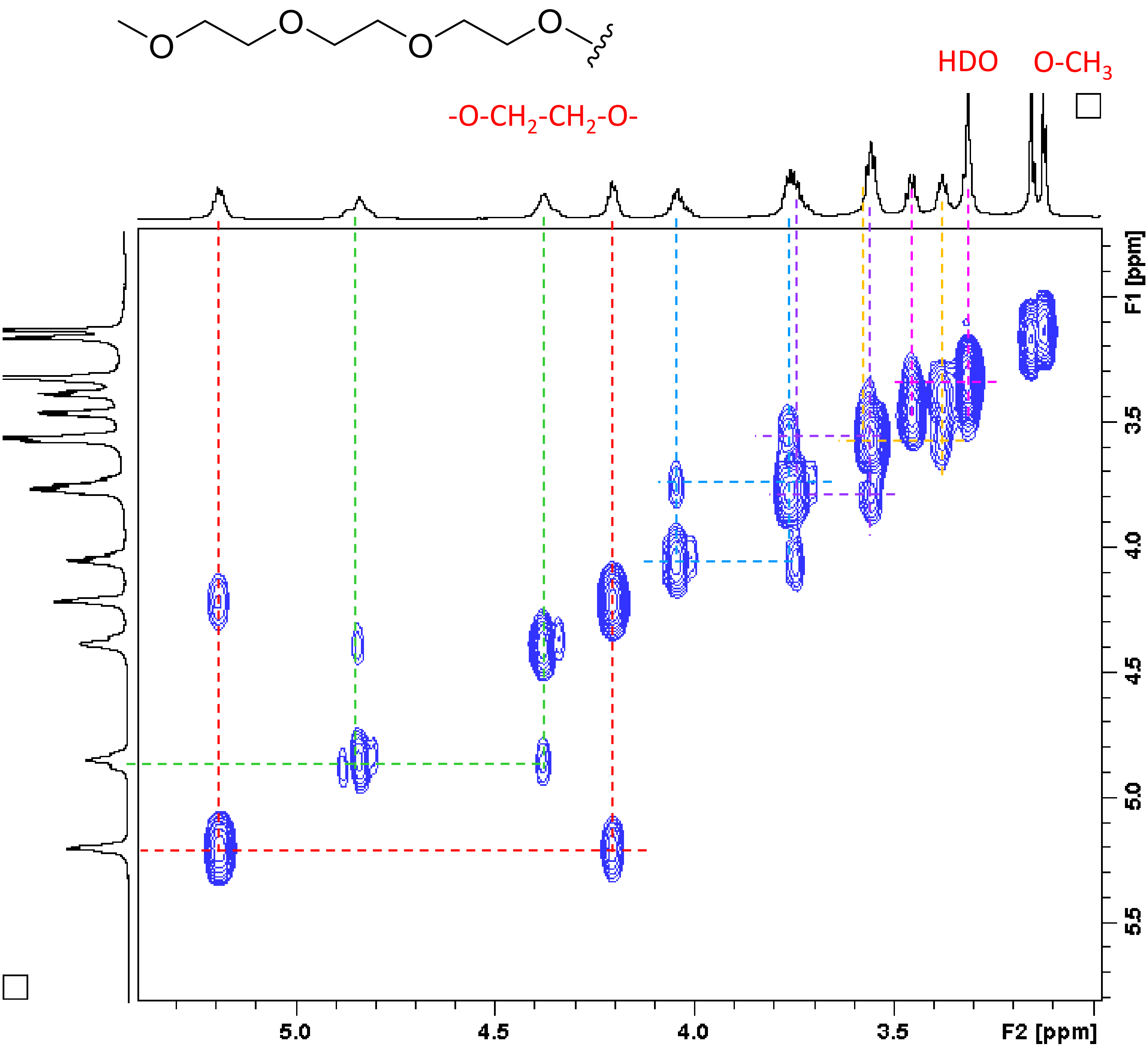

Figure S1 B: $^1H^1H$ COSY spectrum of **Pc1** in DMSO-d_6. Spectral region of TriEG-resonances.

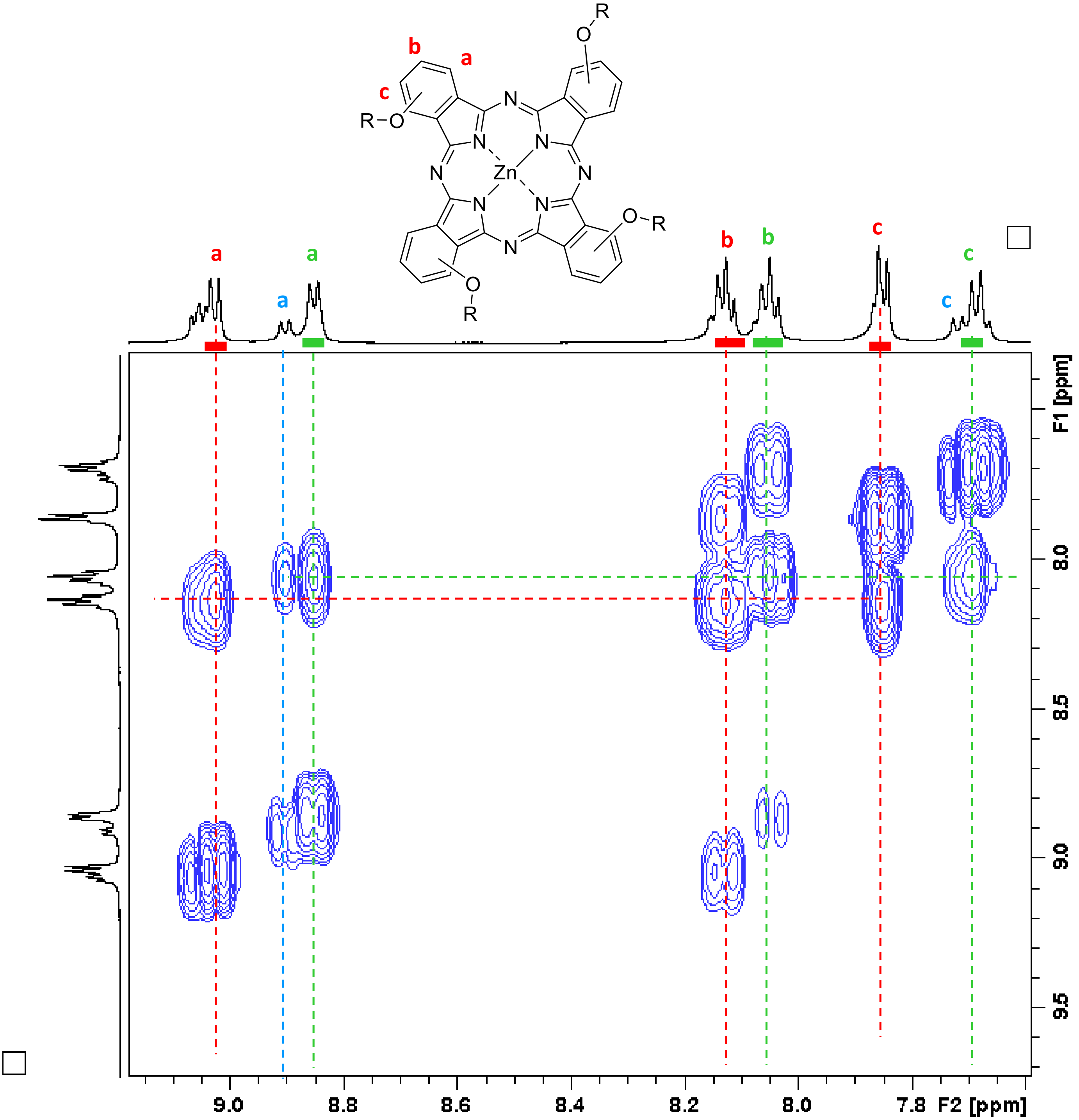

Figure S1 C: $^{1}H^{1}H$ COSY spectrum of **Pc1** in DMSO-d_6. Spectral region of aromatic resonances.

R = 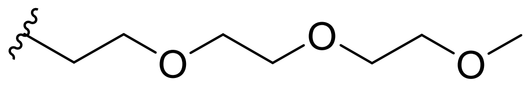

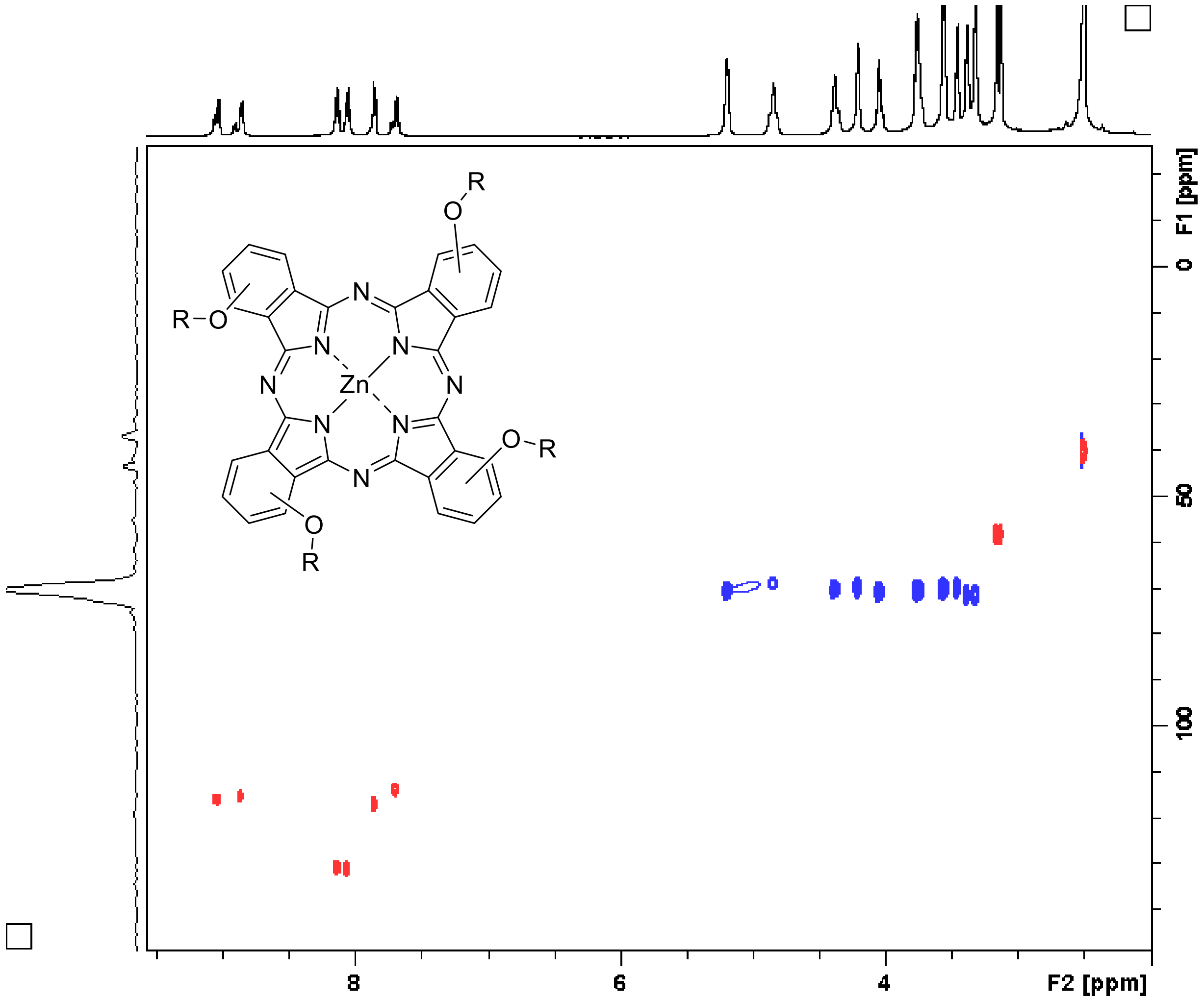

Figure S2: $^{1}H^{13}C$ HSQC spectrum (with multiplicity editing; red: CH, CH_3, blue: CH_2) of **Pc1** in DMSO-d_6.

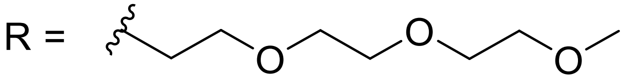

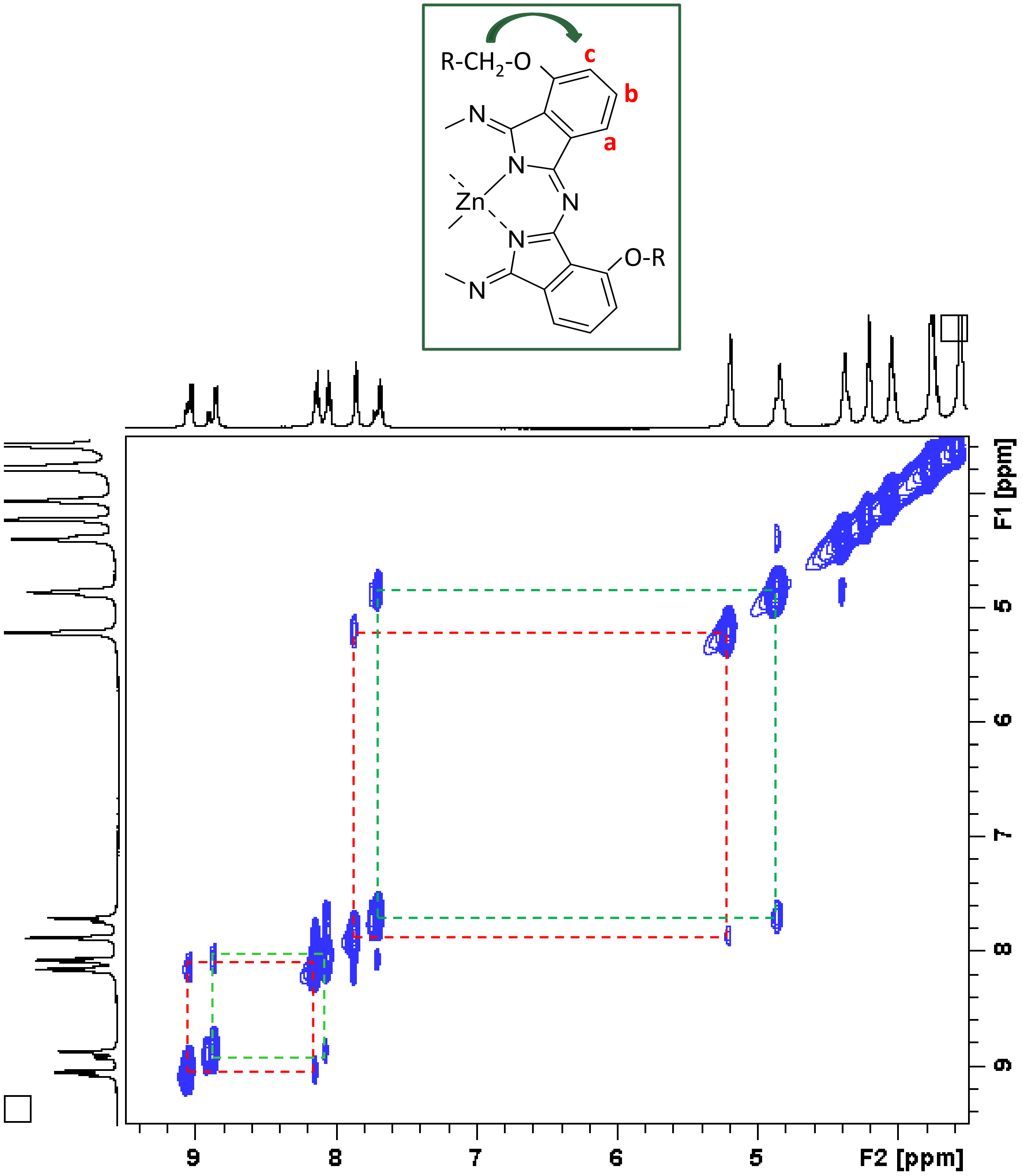

Figure S3: ^{1}H^{1}H NOESY spectrum of **Pc1** in DMSO-d_6 (mixing time T_m = 500 ms).
Two NOE cross peaks between the aromatic H-c protons and the ethylene-glycol protons nearest to the Pc macrocycle are observed indicating the presence of two TriEG-chains with different environments, most likely facing and distant TriEG-chains.

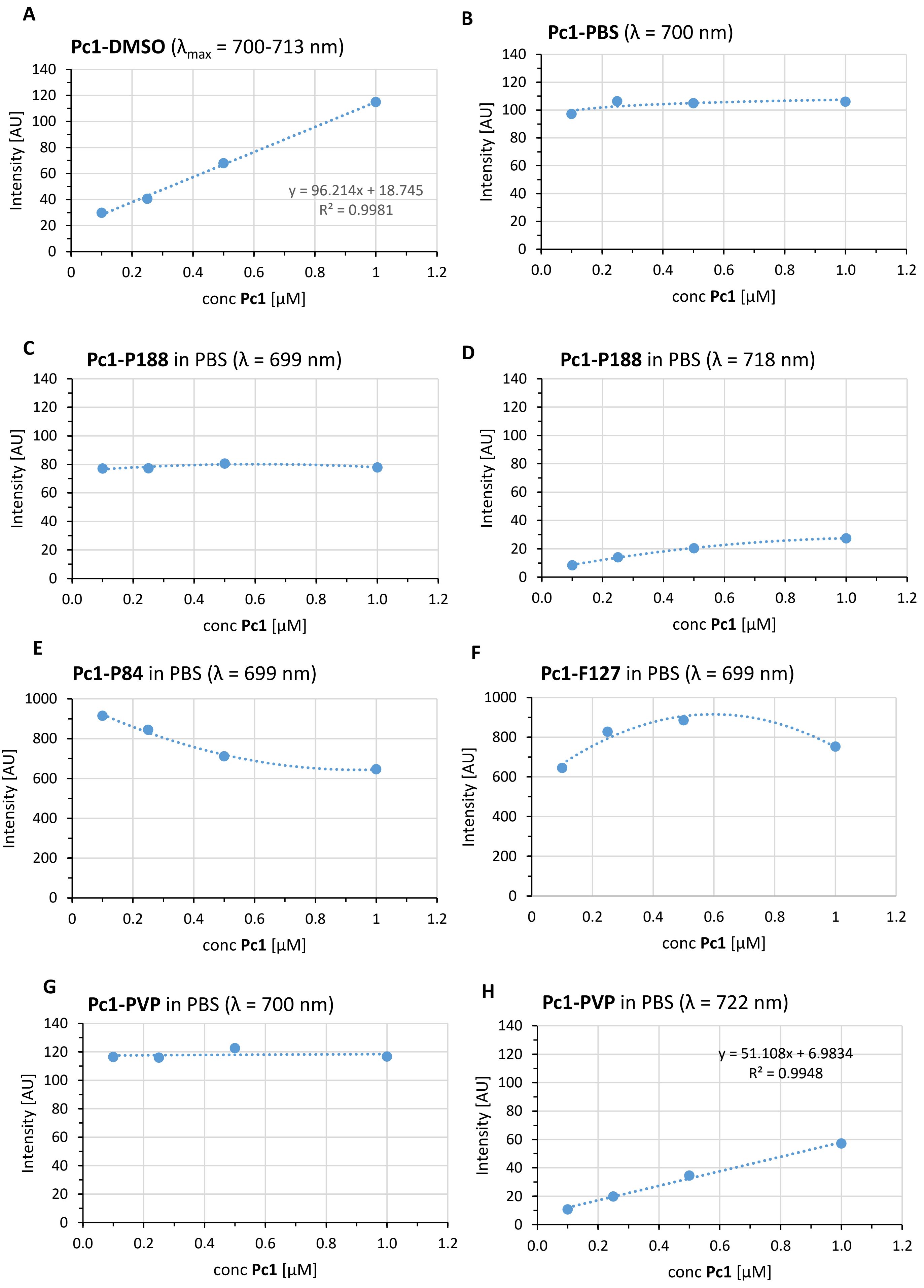

Figure S4: Fluorescence emission at λ_{max} (em) as function of **Pc1** concentration, λexc = 350 nm. (A) **Pc1-DMSO**, (B) **Pc1-PBS**, (C) **Pc1-P188** λ_{max} (699) , (D) **Pc1-P188** λ_{max} (718) , (E) **Pc1-P84**, (F) **Pc1-F127**, (G) **Pc1-PVP** λ_{max} (700) , (H) **Pc1-PVP** λ_{max} (722).

I

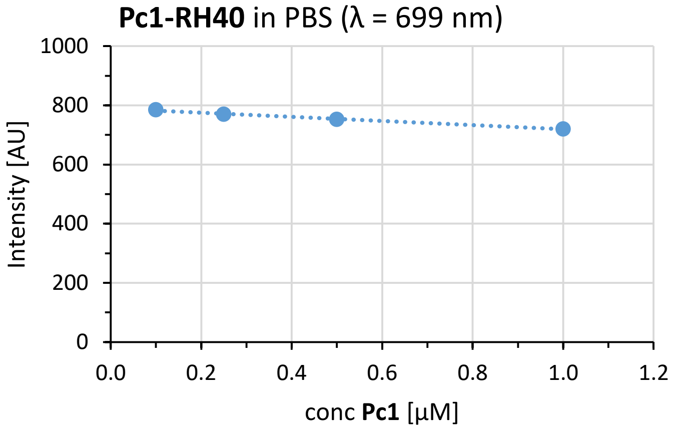

Figure S4 (continued): Fluorescence emission at λ_{max} (em) as function of **Pc1** concentration, λexc = 350 nm. (I) **Pc1-RH40**.

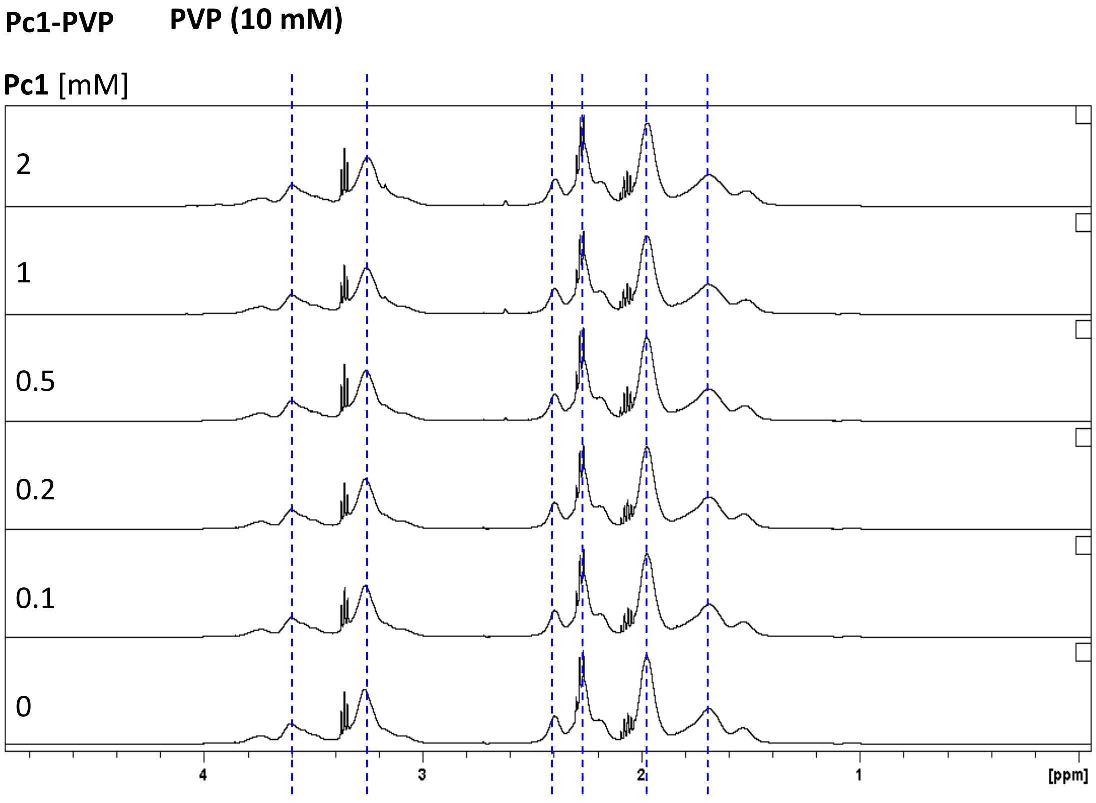

Figure S5: ^{1}H NMR spectra of **Pc1-PVP** (10 mM PVP) in PBS at increasing concentrations of **Pc1**.

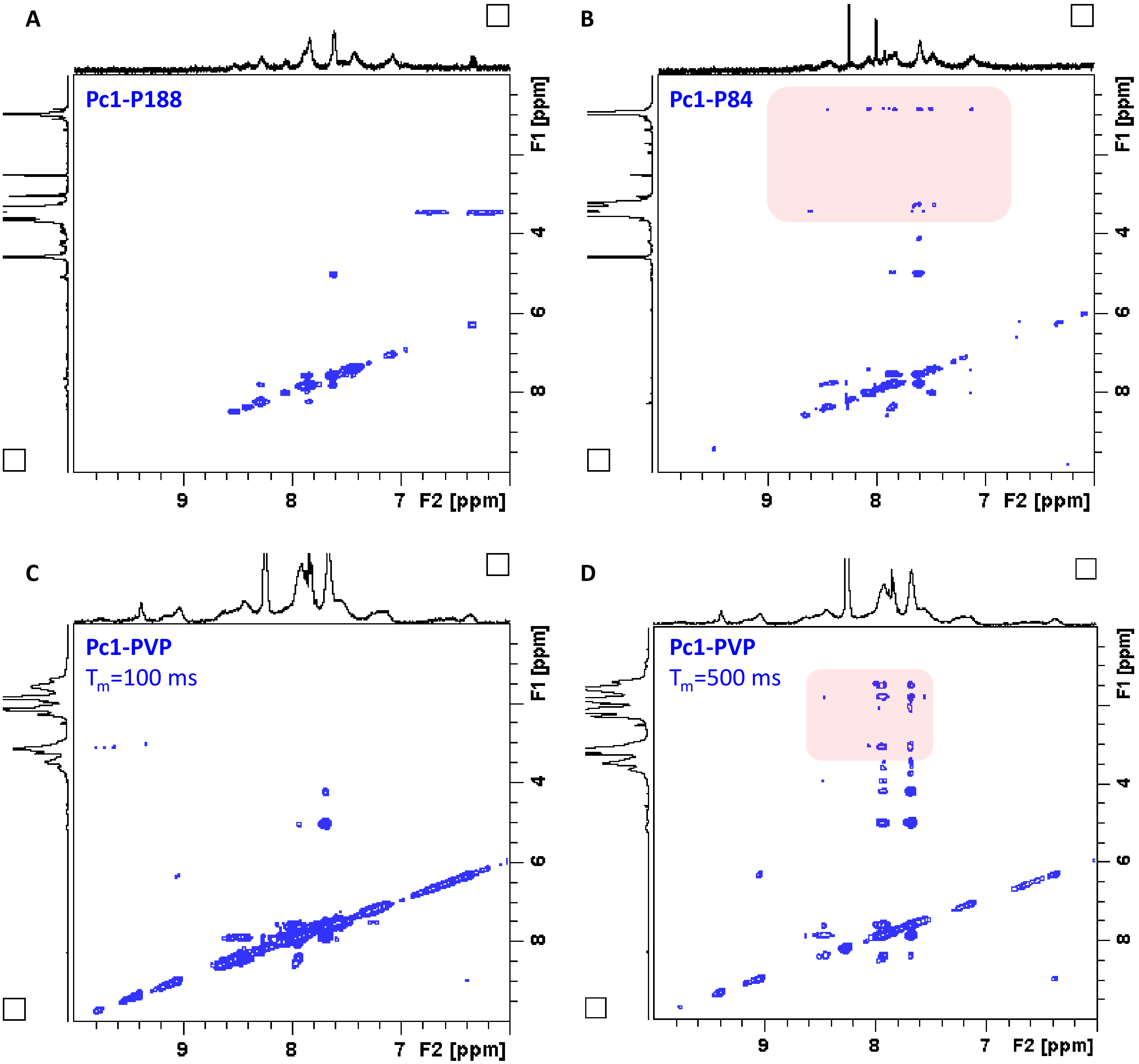

Figure S6: $^1H^1H$ NOESY spectra of (A) **Pc1-P188**, (B) **Pc1-P84**, and (C, D) **Pc1-PVP** at a mixing time T_m of 100 ms (C) and 500 ms (D). All spectra were acquired in PBS (D_2O, pH 7.3) with T_m = 100 ms except (D).
For **Pc1-P84**, weak intermolecular NOE cross peaks between the aromatic protons of **Pc1** and the PPG-methyl and PEG-methylene protons (indicated by the red box) could be observed. For **Pc1-PVP**, only at T_m = 500 ms, intermolecular NOE cross peaks were observed (red box).

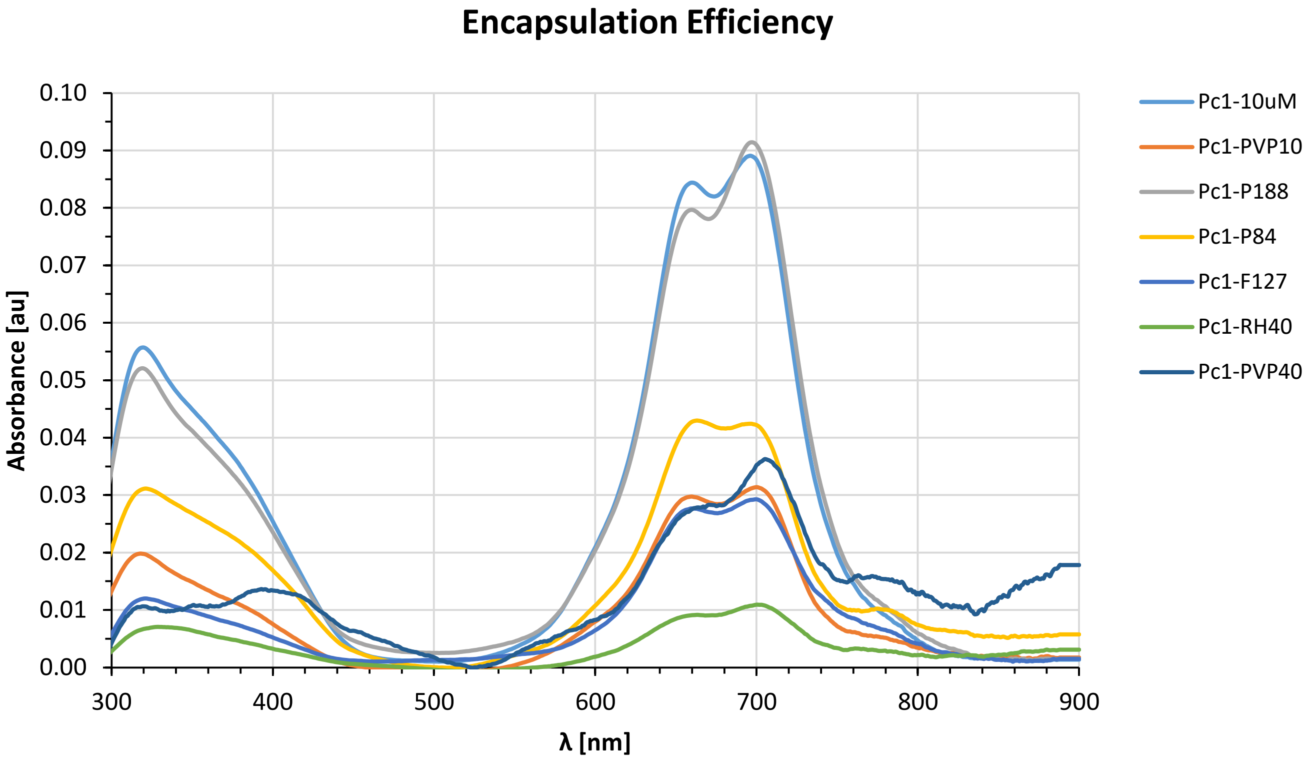

Figure S7: UV-vis absorption spectra of filtrates obtained after ultracentrifugation (MWCO 10 kDa) of **Pc1**-polymer mixtures at a molar ratio of 1 : 10 (1 mM **Pc1**, 10 mM polymer) and of **Pc1-PBS** (1 mM). The filtrates were diluted by a factor of 100 with PBS. For validating the encapsulation efficiency of PVP with a MW of 10 kDa, which is close to the MWCO of the filters, an additional sample was prepared using PVP with a MW of 40 kDa.

Table S1: List of selected Triblock-copolymers (PEG-PPG-PEG) and their synonyms.

Trade name	Supplier	Synonyms	PG units	EG units	MW
Kolliphor P 188	BASF<br>BASF<br>CRODA	Poloxamer P 188<br>Pluronic F 68<br>Synperonic PE/F 68	27-30	154-160	8.4 kDa
Pluronic F 127	BASF<br>BASF<br>CRODA	Poloxamer P 407<br>Kolliphor P 407<br>Synperonic PE/F 127	65	200	12.6 kDa
Synperonic PE/P84	CRODA		43	36	4.2 kDa

Table S2: List of selected PEGylated Castor oils and their synonyms.

Trade name	Supplier	Synonyms	EG units	Chemical name
Kolliphor EL	BASF	Cremophor EL	35	PEG-35 Castor oil; Macrogolglycerol ricinoleate
Kolliphor ELP	BASF<br>CRODA	Cremophor ELP<br>Etocas 35	35	PEG-35 Castor oil; Macrogolglycerol ricinoleate
Kolliphor RH40	BASF<br>CRODA	Cremophor RH40<br>Croduret 40	40	PEG-40 hydrogenated Castor oil; Macrogolglycerol hydroxystearate
Kolliphor RH60	BASF<br>CRODA	Cremophor RH60<br>Croduret 60	60	PEG-60 hydrogenated Castor oil; Macrogolglycerol hydroxystearate
